# Climate Change Vulnerability and Conservation Priorities for Atlantic Forest Palms

**DOI:** 10.1002/ece3.73411

**Published:** 2026-04-08

**Authors:** Júlia Angeli, Daniela Custódio Talora, Gabriela Alves‐Ferreira, Neander Marcel Heming, Eliana Cazetta

**Affiliations:** ^1^ Programa de Pós‐graduação Em Ecologia e Conservação da Biodiversidade, Departamento de Ciências Biológicas Universidade Estadual de Santa Cruz Ilhéus Bahia Brazil; ^2^ Ecological Interactions of Tropical Plants Lab Universidade Estadual de Santa Cruz Ilhéus Bahia Brazil; ^3^ Applied Ecology and Conservation Lab Universidade Estadual de Santa Cruz Ilhéus Bahia Brazil

**Keywords:** Arecaceae, ecological niche models, global warming, life‐history traits, range shift, species distribution, species richness

## Abstract

Climate change is expected to significantly impact biodiversity, causing shifts in palm species distribution. Understanding how climate change affects distribution patterns and identifying traits that influence species' responses may contribute to species conservation. Using ecological niche models and life‐history trait data for 59 palm species of the Brazilian Atlantic Forest, we investigated: (i) how future climate scenarios may affect species richness and climatically suitable areas for Atlantic Forest palms, identifying the most threatened species, and (ii) which traits are associated with gains or losses in the suitable area under climate change. In general, the Atlantic Forest is projected to maintain high levels of palm species richness, although substantial regional losses are expected, particularly along the northern coast and in central areas of the biome, which currently harbor areas of high species richness. Our projections indicate that over 64.4% of palm species will experience contractions in their climatically suitable areas across all future scenarios. Species with restricted distributions are particularly vulnerable, reinforcing the importance of baseline range size as a key determinant of climate change sensitivity. In contrast, life‐history traits were not significant predictors of changes in the area ratio across future scenarios. These findings identify priority regions and species for conservation planning and emphasize the need to protect climatically stable areas, enhance habitat connectivity, and implement restoration strategies to mitigate biodiversity loss in the Atlantic Forest.

## Graphical abstract

Climate change may reduce palm richness in the northern and central Atlantic Forest. Some palms may lose their entire range areas, while others may expand in the future. Range‐restricted palms tend to be more vulnerable to climate change.

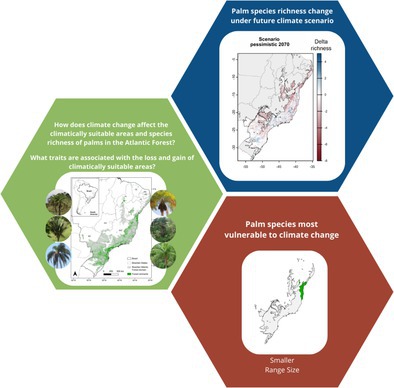

## 1 Introduction

Anthropogenic climate change is already causing profound impacts on biodiversity (Coleman and Bragg 2021), with plants being particularly vulnerable (Parmesan 2006; Walther 2003). According to the latest IPCC report (IPCC 2023), global surface temperatures are projected to continue rising through 2040, with increases ranging from ~1.5°C under low‐emission scenarios to 2.7°C–4.4°C under intermediate and high‐emission scenarios. These changes, including more frequent heat extremes and heavy precipitation events, are expected to drive shifts in plants' distributions as they track suitable climatic conditions, potentially leading to range expansions or contractions and altering ecological interactions (Garcia et al. 2014; Lenoir and Svenning 2015; Sekercioglu et al. 2008; Walther 2010). Such shifts may have cascading effects on ecosystem functioning, particularly in biodiversity‐rich tropical regions (Nunez et al. 2019).

Species' responses to climate change are not random; rather, they may partially depend on traits that influence persistence and expansion under future scenarios (Angert et al. 2011). Species with specific traits may cope better with climate change, whereas the absence of such traits could lead to population declines and even extinctions (Kühn et al. 2021). For example, wider geographic ranges and higher dispersal capacities may help species persist by enhancing their ability to colonize newly suitable areas (Boulangeat et al. 2012; Jenkins et al. 2007). In addition, under high water stress, plant height has been identified as a key trait, with shorter‐statured plants generally showing greater tolerance under climate changes (Kühn et al. 2021). Thus, life‐history traits can help explain how species shift their distribution in response to climate change (Estrada et al. 2016), as demonstrated for other biological groups (Alves‐Ferreira et al. 2022; Mota et al. 2022a). Consequently, projecting future potential distributions of plant species under climate scenarios and understanding how life‐history traits affect these shifts are essential for assessing the impacts of climate change and guiding effective biodiversity conservation strategies.

As a model group in tropical regions, palms (Arecaceae) are particularly valuable for investigating how climate change can influence species' geographic ranges and for identifying life‐history traits associated with range expansions or contractions. This diverse family includes approximately 2500 pantropical species, with South America representing its center of endemism (Pintaud et al. 2008). Palms play a crucial role in maintaining tropical ecosystems (Eiserhardt et al. 2011). They are often considered keystone species, providing fruits for a wide variety of frugivorous animals that are essential for seed dispersal (Galetti et al. 2001; Zona and Henderson 1989) and offering inflorescences for a diverse array of pollinators (Henderson 2024). In the Atlantic Forest, one of the world's most threatened and climate vulnerable biomes (Bellard et al. 2014), palms are highly diverse, with 54 species endemic to the region (Pintaud et al. 2008). This biome also harbors one of the highest levels of tree species richness globally (Myers et al. 2000), highlighting the ecological importance of palms in maintaining forest structure and interactions. Climatic factors, particularly water availability and temperature, are critical determinants of palm distributions (Bjorholm et al. 2005; Blach‐Overgaard et al. 2009; Kreft et al. 2006; Svenning et al. 2008). Consequently, palms and the ecosystem services they provide are affected by climate change, which may lead to either expansion or contraction of their geographic ranges under future scenarios (Blach‐Overgaard et al. 2015; Costa et al. 2022; Vaz and Nabout 2016; Walther et al. 2007; Sales et al. 2021).

Although palms are widely recognized as keystone species in tropical ecosystems and have been the focus of numerous ecological and biogeographical studies, few macroecological analyses have linked traits to range shifts under climate change. Their high ecological and economic importance, combined with sensitivity to climatic gradients, makes them an ideal system for trait‐based assessments of species' responses to future climate scenarios, especially in vulnerable biomes such as the Atlantic Forest. Here, we address this gap by combining life‐history trait data with Ecological Niche Models (ENMs) to assess how traits may influence changes in the distribution area of palms under future climate scenarios. Specifically, we aimed to (a) assess how future climate scenarios may affect species richness and climatically suitable areas for Atlantic Forest palm species, identifying the most threatened species, and (b) evaluate which palm traits are associated with the greatest gains or losses of suitable areas under future climate change scenarios. It is expected that palms with (i) restricted geographical ranges and (ii) forest interior species, primarily found in closed‐canopy forests, will be more vulnerable to climate change, showing a decrease in the climatically suitable areas under future climate scenarios (Blach‐Overgaard et al. 2015; Lee et al. 2015). Furthermore, species expected to gain suitable areas in future scenarios are predicted to exhibit traits such as (iii) shorter stem height and (iv) larger fruits (Table 1).

**TABLE 1:** Predicted relationships between palm life‐history traits/ranges and projected changes in the suitable climatic area under future climate scenarios in the Atlantic Forest.

Trait/range	Predicted response under climate change	Physiological/ecological explanation	References
(i) Restricted geographical range	Decrease in the suitable area	Species with small ranges have limited capacity to track suitable climates, making them more vulnerable.	Lee et al. 2015
(ii) Forest interior species (closed‐canopy)	Decrease in the suitable area	Dependence on shaded, humid microhabitats makes them highly sensitive to warming and forest loss.	Blach‐Overgaard et al. 2015
(iii) Shorter stem height	Increase in the suitable area	Reduces water loss through the stem, enhancing drought and heat tolerance.	Cássia‐Silva et al. 2022
(iv) Larger fruits	Increase in the suitable area	Larger seeds improve establishment in stressful conditions and enable longer dispersal distances via frugivores.	Göldel et al. 2015; Westoby et al. 1996; Jenkins et al. 2007

## 2 Methods

### 2.1 Study Area

**FIGURE 1:**
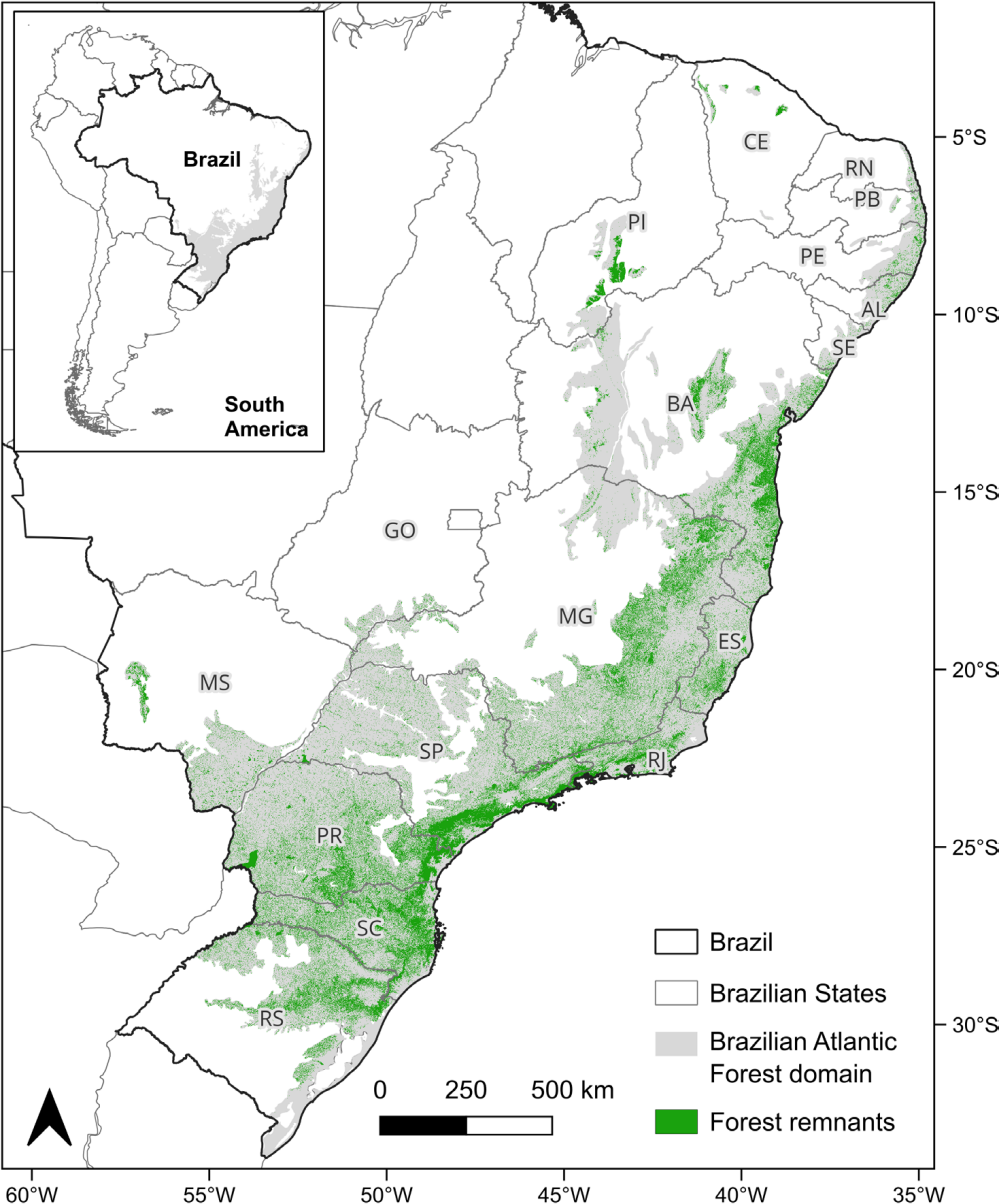
Map of the Brazilian Atlantic Forest, showing the current forest cover. Brazilian states within the Atlantic Forest domain are shown with their abbreviations: Alagoas (AL); Bahia (BA); Ceará (CE); Espírito Santo (ES); Goiás (GO); Mato Grosso do Sul (MS); Minas Gerais (MG); Paraíba (PB); Paraná (PR); Pernambuco (PE); Piauí (PI); Rio de Janeiro (RJ); Rio Grande do Norte (RN); Rio Grande do Sul (RS); Santa Catarina (SC); São Paulo (SP); and Sergipe (SE).

This study was conducted in the Brazilian Atlantic Forest domain (Figure 1), one of the world's main biodiversity hotspots, characterized by high species richness, elevated levels of endemism, and only a small portion of its original forest cover remaining (Myers et al. 2000). The biome spans a wide latitudinal range along the Brazilian coast (from 3° S to 33° S and 35° W to 60° W), covering almost 15% of the Brazilian territory (de Muylaert et al. 2018; Ribeiro et al. 2009). It also exhibits considerable climatic variation, with annual rainfall ranging from 1000 to 4200 mm, and encompasses a variety of habitats along altitudinal and environmental gradients (Ribeiro et al. 2011; Peres et al. 2020). Since colonization in the 16th century, the biome has undergone intense transformations due to human activity, leading to forest deforestation and fragmentation, which have significantly impacted its biodiversity (Joly et al. 2014). Currently, forest vegetation in the Atlantic Forest covers only 22.9% of its original area, reflecting a significant loss of habitat (Vancine et al. 2024). The Atlantic Forest is legally protected under Brazilian law (Brazil 2006), and it is considered a priority area for conservation due to its high biodiversity and endemicity, making it particularly relevant for studies on species responses to climate change.

### 2.2 Palm Data

The dataset used in this study was developed and published elsewhere (Angeli et al. 2025). For clarity and reproducibility, we summarize below the main data sources and processing steps used in this analysis. Briefly, the dataset initially included 78 palm species based on the official treatment of Arecaceae in the *Flora e Funga do Brasil* project and the study by Cerqueira et al. (2023), of which 54 are endemic to the Atlantic Forest (Pintaud et al. 2008). This list was expanded with 10 additional species reported by Bello et al. (2017), totaling 88 species. All species were subsequently checked for native status within the Atlantic Forest using *Flora e Funga do Brasil*, Cerqueira et al. (2023), and Bello et al. (2017). After excluding non‐native, exotic, invasive, and naturalized species within the biome, 83 palm species remained in the dataset for occurrence record download.

Occurrence records were gathered from the Global Biodiversity Information Facility (GBIF; www.gbif.org), a platform that consolidates data from various sources and is commonly used for predicting species distribution (Heberling et al. 2021). Additional coordinates were also obtained from the databases: SpeciesLink (https://www.specieslink.net), Integrated Digitized Biocollections (iDigBio; https://www.idigbio.org), and iNaturalist (https://www.inaturalist.org). The nomenclature and synonyms used follow the *Flora Brasileira: Arecaceae (Palmeiras)* (Lorenzi et al. 2010) and Cerqueira et al. (2023). After obtaining the data, we performed a quality control on the occurrences to clean the coordinates by removing inaccurate records, including those with incomplete coordinates and those corresponding to municipality centroids (Zizka et al. 2019). Additionally, we removed occurrences that were less than 10 km apart from their nearest neighbors through the *spThin* optimization algorithm (Aiello‐Lammens et al. 2015), improving ecological niche model performance (Boria et al. 2014). Only species with at least five occurrence records were retained for subsequent analyses. The final cleaned dataset comprised 2708 occurrence records for 59 palm species (Table S1).

For each palm species, we used key plant traits (Table 2) commonly associated with species distribution and responses to anthropogenic changes: stem height, stem diameter, fruit length, and fruit width (Bjorkman et al. 2018; Cornelissen et al. 2003; Göldel et al. 2015; Westoby 1998). These data were obtained from the global palm trait database, PalmTraits 1.0 (Kissling et al. 2019). Additional information for certain species was gathered from *Flora Brasileira: Arecaceae (Palmeiras)* (Lorenzi et al. 2010), the *Flora e Funga do Brasil* project (Arecaceae in Flora e Funga do Brasil 2024), and other literature sources (Table S2). Missing trait values were estimated at the genus level when appropriate, with all imputation procedures for both trait data and habitat classification described in detail by Angeli et al. (2025).

**TABLE 2:** Traits of 59 palm species in the Atlantic Forest, including mean, maximum, and minimum values.

Traits	Type	Mean (max, min)
Habitat	Categorical (Open area, Forest interior)	
Maximum stem height	Continuous	9.25 cm (0 e 40)
Maximum stem diameter	Continuous	18.67 cm (0 e 66)
Mean fruit length	Continuous	2.98 cm (0.6 e 12.5)
Mean fruit width	Continuous	2.12 cm (0.55 e 6)

### 2.3 Climate Data

We obtained 19 bioclimatic variables from the WorldClim v2.1 platform (Fick and Hijmans 2017) at a resolution of 2.5 arc‐min (~4.5 km^2^) (Table S3). This resolution is widely used in species distribution studies and represents a balance between computational feasibility and spatial detail. These variables are derived from precipitation and temperature measurements for the period of 1970–2000 (hereafter known as baseline). We also obtained future climate projections for two periods, 2050 (2041–2060) and 2070 (2061–2080), considering three Global Climate Models (GCMs): IPSL‐CM6A‐LR, MIROC6, and MRI‐ESM2‐0, and two Shared Socio‐economic Pathways (SSPs): 245 and 585. The selected GCMs were chosen due to their good performance in global evaluations, including South America, particularly regarding frequency and persistence errors (Cannon 2020), and their demonstrated good performance in climate projections for the Atlantic Forest (Mota et al. 2022b). We calculated a weighted average to combine the results of the three GCMs using the R function “consensus_scn_b” implemented in the *ENMwizard* R package (Heming et al. 2019). The SSP2‐4.5 represents an intermediate emissions pathway, whereas SSP5‐8.5 represents a high‐emissions pathway (O'Neill et al. 2014). This combination resulted in four future scenarios (two time periods × two SSPs), covering a broad range of potential climatic conditions. We calculated a correlation matrix to reduce collinearity issues among the bioclimatic variables, using Pearson correlation coefficients with a cutoff value of 75% and the “select_vars” function from the *ENMwizard* R package (Heming et al. 2019). We selected only the bioclimatic variables that exhibited correlation below the cutoff limit. The selected climatic variables for each species are listed in Table S3.

### 2.4 ENMs

ENMs estimate the relationship between environmental variables and known species occurrences, enabling geographic projections of species distributions over time (Elith et al. 2011). We organized the description of our methods according to the ODMAP protocol (Overview, Data, Model, Assessment, and Prediction; Zurell et al. 2020). Below we provide a concise summary, while full details of each modeling step are presented in the Supporting Information. To model the climatic niches of 59 palm species, we used the MaxEnt algorithm (version 3.4.1; Phillips et al. 2017) implemented through the *ENMwizard* package (Heming et al. 2019). MaxEnt requires only occurrence data and bioclimatic variables (Phillips et al. 2006) and is widely recognized for its high performance and accuracy, even when applied to small samples (Hernandez et al. 2006). In addition, previous studies focusing on palms have also achieved good performance using MaxEnt (Blach‐Overgaard et al. 2009, 2010). The calibration area was created with a 1.5° buffer around the minimum convex polygon (MCP) of all occurrences. The models were fitted using options for Feature classes (FC) and Regularization multipliers (RMs), including all combinations of the following classes: linear (L), product (P), and quadratic (Q), along with RM values (ranging from 0.5 to 5, with increments of 0.5), resulting in 70 models per species. These two parameters are important in defining the complexity of MaxEnt models, where RMs control for regulating overfitting, and FCs represent transformations of the original bioclimatic variables (Muscarella et al. 2014).

To validate and assess the predictive capacity of the models, we applied two methods of geographical partitioning on the occurrence records: block or jackknife, using the “ENMevaluate_b” function from the *ENMwizard* package (Heming et al. 2019). The jackknife partitioning method was used for species with fewer than 15 occurrence records (Shcheglovitova and Anderson 2013). This method involves removing one point from the dataset at a time and building a new model without the removed point (Pearson et al. 2007). In contrast, the block method divides the occurrence points into four spatially independent blocks, performing four iterations in which one block is used to evaluate the model, while the others are used for calibration (Roberts et al. 2017).

We constructed a consensus model for each species from the top 10% of MaxEnt models based on the criteria of the lowest omission rate (OR) and the highest mean area under the curve (AUC) (Boria et al. 2017). The performance metrics of the model (AUC and OR) were calculated using the *ENMeval* package (Muscarella et al. 2014). The potential continuous distributions of each species were then converted into a binary vector (climatically suitable and unsuitable areas) by applying a threshold that excludes 10% of the occurrence points with the lowest environmental suitability values (Liu et al. 2005). Then, we projected them into future climate scenarios (two time periods × two SSPs). Subsequently, to correct the overestimation produced by ecological niche models, we created a MCP around all filtered occurrence records within the study area and used this polygon to refine the binary models. This adjustment ensured that the resulting maps were free from overprediction. Buffers were not applied to these maps, as the limited dispersal capacity of palms (Blach‐Overgaard et al. 2010; Carrete et al. 2022; Tella et al. 2020) would not affect the pixel size of each species' map.

### 2.5 Data Analysis

Species richness was calculated by summing the binary species maps for each climatic scenario (baseline and future—2050 SSPs 245 and 585 and 2070 SSPs 245 and 585) through the “rast.sr” function from the *phyloraster* R package (Alves‐Ferreira et al. 2024). Subsequently, we computed the difference between species richness in the baseline and future scenarios (delta) using the “delta.grid” function from *phyloraster* (Alves‐Ferreira et al. 2024). Positive “delta richness” values indicate species gains in the future, while negative values denote species losses. Finally, we calculated the change in the suitable area for each species in each future scenario relative to the baseline scenario using the following formula:

$$\text{Area change}=\frac{\text{Future climatically suitable area}}{\text{Baseline suitable area}}$$

In this ratio, values equal to 1 indicate stability (i.e., no change in suitable area), values greater than 1 indicate an expansion of the suitable area, values between 0 and 1 indicate a contraction, and a value of 0 indicates a complete loss of the suitable area.

The relationship between species traits and projected changes in distribution may be influenced by shared evolutionary history, as closely related species tend to share similar traits (Losos 2008; Angert et al. 2011). Such phylogenetic non‐independence can bias cross‐species statistical analyses if not properly accounted for. To assess the presence of phylogenetic signal among traits, we used Fritz and Purvis' D statistic (Fritz and Purvis 2010) for categorical traits (e.g., habitat) and Pagel's λ (Lambda) (Pagel 1999) for continuous traits (e.g., maximum stem height, maximum stem diameter, average fruit length, average fruit width). These metrics were calculated using the “phylo.d” function from the *caper* R package (Orme et al. 2025) and “phylosig” function from the *phytools* R package (Revell 2024), respectively. For D statistics, values equal to 1 indicate that a trait is randomly distributed across the phylogeny, values around 0 indicate a distribution consistent with a Brownian motion model, and values below 0 indicate stronger phylogenetic clustering. For λ, a value of 1 indicates a phylogenetic pattern consistent with Brownian motion, values between 0 and 1 indicate intermediate phylogenetic signal, and a value of 0 indicates no phylogenetic signal (Pearse et al. 2025).

Because several traits showed a phylogenetic signal (Table S4), we incorporated phylogenetic structures into our subsequent analyses using Phylogenetic Generalized Least Squares (PGLS) models to assess the influence of predictor variables on the response variable, which is the ratio of change in the suitable area for each future climate scenario. The phylogeny was based on the maximum clade credibility tree calculated using the “maxCladeCred” function from the *phangorn* R package (Schliep 2011), derived from the 1000 Constraints trees of the species‐level palm phylogeny from Faurby et al. (2016). The tree was pruned to include only the 59 species in our dataset, and missing species were added using the “phylo.maker” function from the *V.PhyloMaker2* R package (Jin and Qian 2022). The resulting tree is available in the Newick format and can be visualized in Figure S1.

We used the “vif.cor” function from the *usdm* R package (Naimi et al. 2014) to check the collinearity among predictor variables (habitat, maximum stem height, maximum stem diameter, average fruit length, average fruit width, and baseline distribution area) with a threshold of 0.5. Variables with collinearity above this threshold were excluded. Thus, we performed the PGLS with the following variables: Habitat, maximum stem height, average fruit width, and baseline distribution area.

We assessed the additive effects of predictor variables on the ratio between future and baseline suitable areas using the “pgls” function from the *caper* R package (Orme et al. 2025). We built four global models (one for each response variable) that included all predictor variables. From the global models, we generated all possible models by removing one trait at a time until reaching the null model, using the “dredge” function from the *MuMIn* R package (Barton 2024), resulting in 16 models for each response variable (Tables S5–S9). The best models were selected based on the lowest AICc (Burnham and Anderson 2004). The homoscedasticity and normality of the model residuals were checked through visual inspection and normality tests.

## 3 Results

### 3.1 Richness Patterns and Climatically Suitable Area

The ENMs showed good performance, with an average AUC of 0.86 (range: 0.7–0.98) and an OR of 0.18 (range: 0–0.5). However, 14 species had moderate fits, with AUC values ranging from 0.5 to 0.66.

**FIGURE 2:**
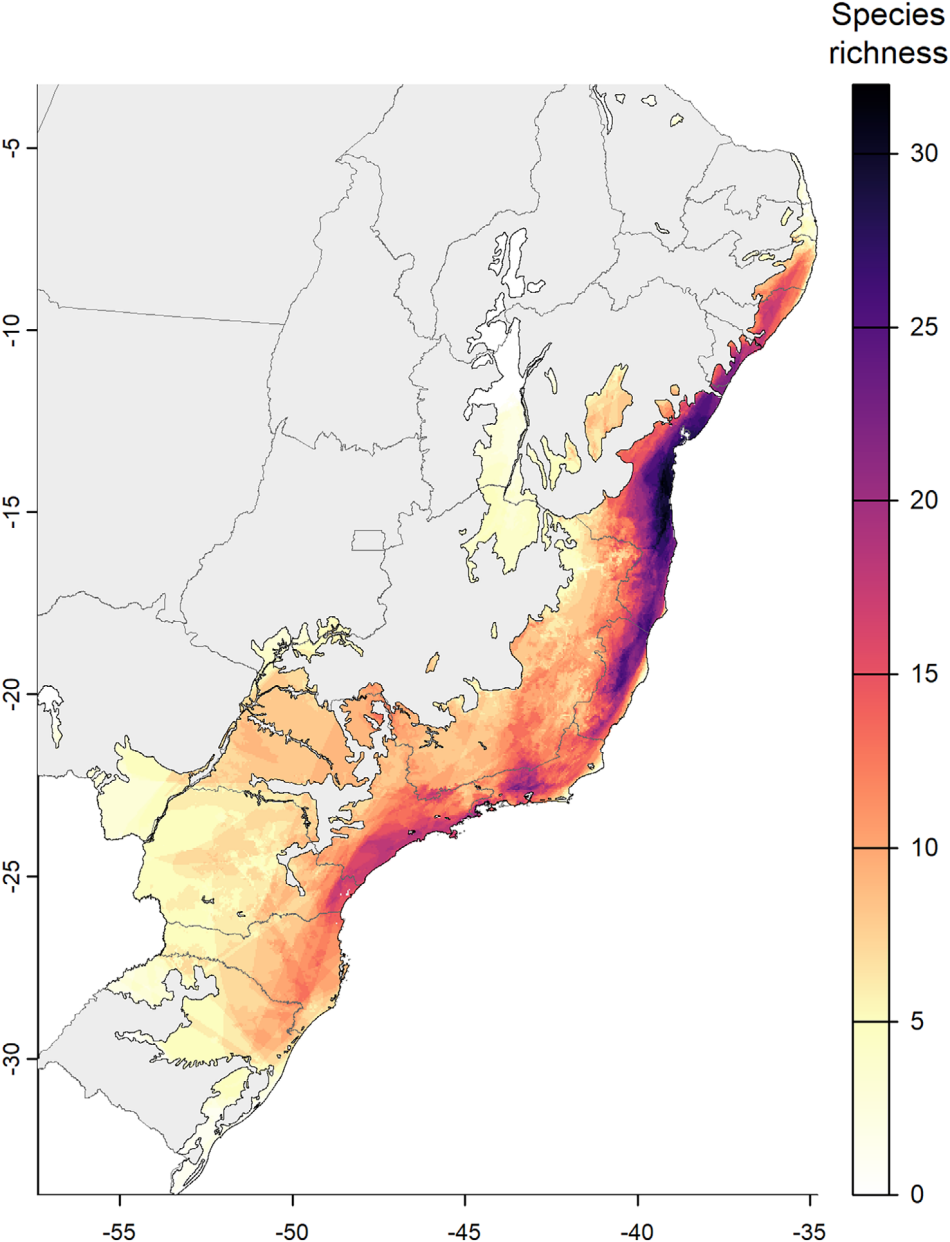
Species richness—map illustrating the variation in palm species richness in the baseline scenario, with darker purple areas representing regions of higher species richness and lighter orange and yellow shades indicating areas with fewer species. White regions highlight areas where no palm species are present.

**FIGURE 3:**
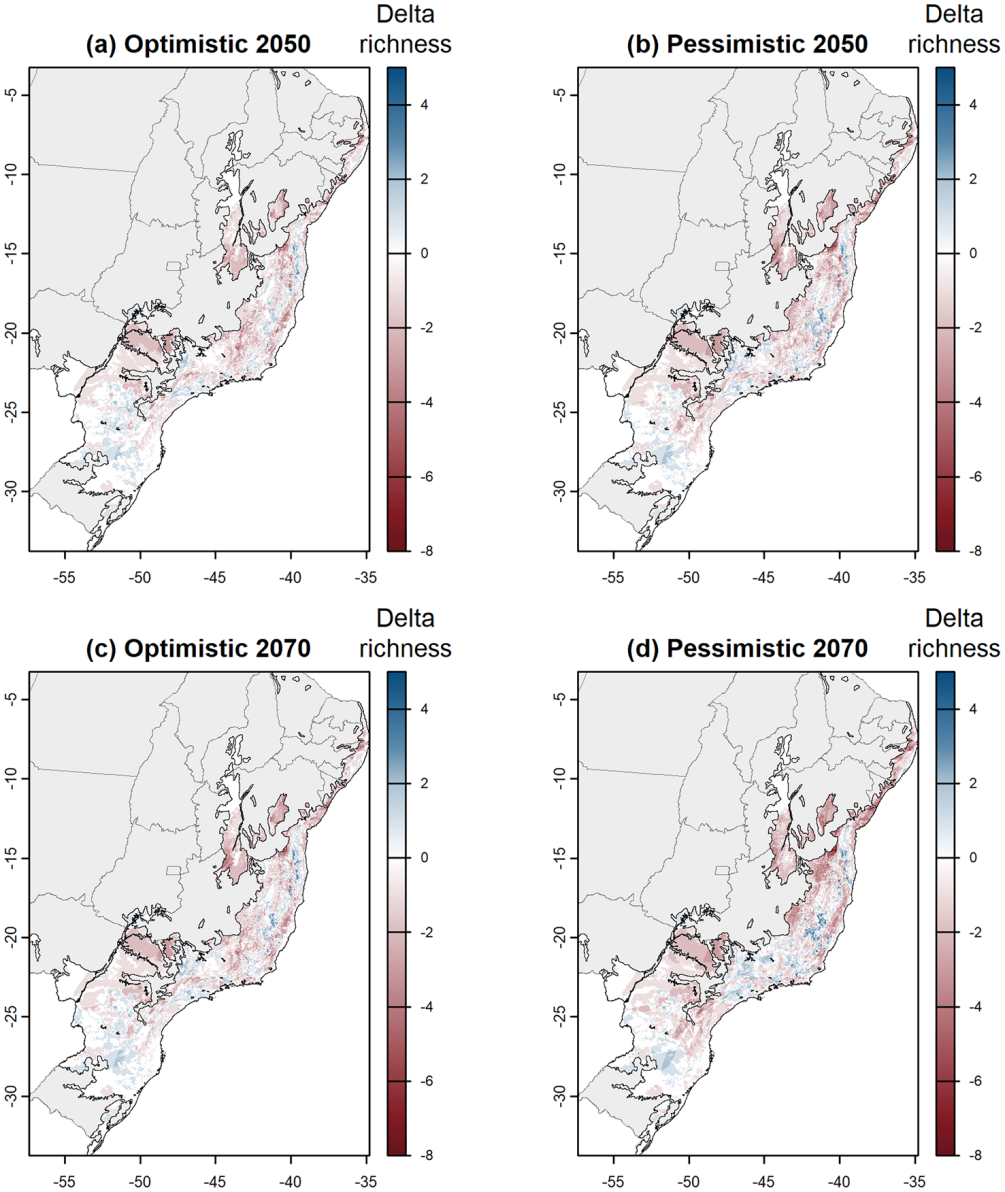
Delta species richness—the maps illustrate the variation in species richness between the baseline and future climate scenarios. Delta richness for (a) optimistic 2050 scenario; (b) pessimistic 2050 scenario; (c) optimistic 2070 scenario; (d) pessimistic 2070 scenario. Blue areas represent regions with an increase in species richness, red areas indicate regions with a decrease, and white areas denote regions where species richness remains unchanged compared to the baseline scenario.

Most Atlantic Forest areas are predicted to maintain species richness (Figure 2) in future scenarios. However, significant losses are also anticipated. Our results indicated that some regions of the Atlantic Forest are projected to lose species under future climate change scenarios (Figure 3a–d), with the states of Bahia (BA), Sergipe (SE), Alagoas (AL), and Pernambuco (PE) being the most affected. The greatest losses in palm species richness were predicted for the northern coast of the Atlantic Forest and central areas of the biome—some regions that currently exhibit the highest levels of species richness—with losses of up to eight species. Conversely, some regions are projected to experience gains in species richness, with increases of up to six species. The largest gains are predicted for certain areas in the southeastern and southern Atlantic Forest, including parts of Minas Gerais (MG), Rio de Janeiro (RJ), São Paulo (SP), Santa Catarina (SC), and Rio Grande do Sul (RS).

The ratio of suitable area changes for palm species ranged from zero to 2.01 (Table S8). By 2050, 42 out of 59 species (71.2%) are projected to experience a decrease in climatically suitable areas under both the optimistic and pessimistic scenarios. By 2070, 42 species (71.2%) are expected to reduce their potential distribution under the optimistic scenario, and 38 species (64.4%) under the pessimistic scenario (ratio < 1). Conversely, 17 species (28.8%) are projected to experience gains in suitable areas under both 2050 scenarios and the 2070 optimistic scenario, whereas 21 species (35.6%) are expected to gain suitable areas under the 2070 pessimistic scenario (ratio > 1).

Several species are projected to undergo substantial losses (Figure S2a–d). *Syagrus lorenzoniorum* Noblick & Lorenzi is projected to undergo extreme contractions in suitable areas, losing more than 99% of its climatically suitable range under the pessimistic scenarios in both 2050 and 2070 (ratio < 0.01). *Attalea apoda* Burret is among the most affected, losing more than 90% of their suitable area under multiple scenarios (pessimistic projections for 2050 and 2070). *Acrocomia intumescens* Drude and *Attalea oleifera* Barb.Rodr. are also highly vulnerable, with reductions surpassing 80% under the 2070 pessimistic scenario. Economically important species in the Atlantic Forest are also affected: *Euterpe edulis* Mart. (juçara palm) is projected to lose approximately 10%–15% of its suitable area under pessimistic scenarios, whereas *Syagrus romanzoffiana* (Cham.) Glassman shows high stability, with only minor losses (< 5%) across all projections. In contrast, several palm species are projected to expand their climatically suitable area under all future scenarios (ratio > 1). These include *Geonoma rubescens* H.Wendl, *Acrocomia aculeata* (Jacq.) Lodd. ex Mart., *Astrocaryum aculeatissimum* (Schott) Burret, *Bactris ferruginea* Burret, *Bactris vulgaris* Barb.Rodr., *Bactris setosa* Mart., *Bactris glassmanii* Med.‐Costa & Noblick ex A.J.Hend., *Bactris bahiensis* Noblick ex A.J.Hend., *Desmoncus orthacanthos* Mart., *Desmoncus polyacanthos* Mart., *Attalea funifera* Mart, *Attalea humilis* Mart, *Syagrus macrocarpa* Barb.Rodr., *Syagrus ruschiana* (Bondar) Glassman, *Butia catarinensis* Noblick & Lorenzi, *Butia paraguayensis* (Barb.Rodr.) Bailey, and *Allagoptera arenaria* (Gomes) Kuntze (Figure S2a–d). This group includes *Attalea funifera* , used for piassava fiber, which is expected to benefit from future climatic conditions, increasing its potential distribution by 18%–41%.

### 3.2 Palm Traits

**FIGURE 4:**
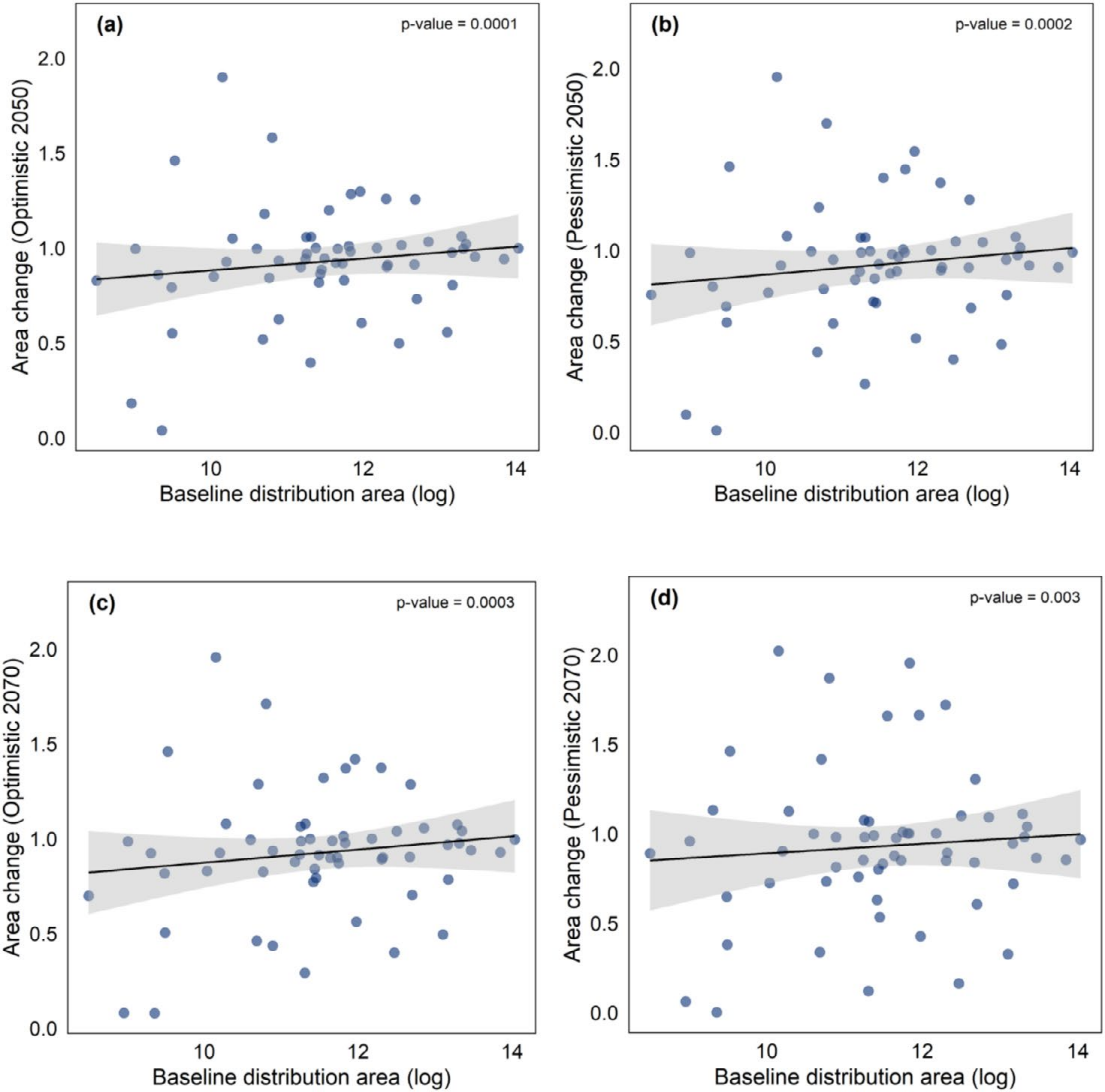
Effects of the baseline distribution area (a–d) on area change across future climate scenarios for 59 Atlantic Forest palm species. Graphics show the relationship between baseline distribution area and area change across future climate scenarios. Each point represents one of the 59 species, with variation shown for those with more restricted or widely distributed areas. Significance was assessed using phylogenetic generalized least squares (PGLS) regression models, with shaded areas around the regression lines representing the standard error of the estimates. The *p*‐value for each response variable is provided.

We found that the baseline distribution area significantly explained projected changes in climatically suitable areas across future climate scenarios. For each scenario, the models with the lowest AICc included the baseline distribution area as a significant predictor (*p* < 0.05, Table 3), supporting our hypothesis. Specifically, palm species with more restricted distribution areas exhibited lower area ratios, indicating a reduction in their suitable habitat across all future scenarios (Figure 4, Table 3). Our variables maximum stem height, fruit width, and habitat were not included in the best model in any scenario.

**TABLE 3:** Model selection results based on the lowest AIC for each response variable. Coefficients of the variables included in each model are presented, along with significance levels.

	Optimistic 2050	Pessimistic 2050	Optimistic 2070	Pessimistic 2070
Intercept	−0.157	−0.251	−0.204	−0.162
Habitat	n.a.	n.a.	n.a.	n.a.
Baseline distribution area (log)	0.092 ***	0.099 ***	0.095 ***	0.090 **
Average Fruit Width	n.a.	n.a.	n.a.	n.a.
Max Stem Height	n.a.	n.a.	n.a.	n.a.
Residual standard error	0.101	0.115	0.114	0.137
Degrees of freedom	57	57	57	57
Adjusted R‐squared	0.220	0.201	0.190	0.122
F‐statistic	17.39	15.61	14.68	9.06
*p*‐value (residuals Normality test)	0.391	0.292	0.241	0.222
Model weight	0.354	0.339	0.382	0.301

*Note:* ***p < 0.001, **p < 0.01. n.a. indicates that the variable was not included in the best model.

## 4 Discussion

Our projections for the Atlantic Forest reveal substantial changes in palm species richness across the biome, with gains and losses driven by climate‐induced shifts in climatically suitable areas. Pessimistic scenarios indicate potential losses of climatically suitable areas for some species, while others may expand their ranges under future climate conditions. Our results also show that palms with restricted distributions are particularly susceptible to climate change, resulting in greater losses of suitable areas.

Future climate projections suggest a complex reorganization of palm species in the Atlantic Forest, with spatially dynamic patterns of species loss and gains affecting regions of the biome unevenly. Areas that currently harbor the highest palm species richness, such as the northern coastal and central portions of the biome, are projected to experience the greatest losses. In contrast, other regions show more heterogeneous responses, with localized gains and losses. These results are consistent with broader evidence showing that climate change is already reshaping plant distributions worldwide (Feeley and Rehm 2012; Kelly and Goulden 2008) and highlighting a complex structural reorganization of the Atlantic Forest in response to climate change already seen for other angiosperms (Zwiener et al. 2017). These findings align with previous projections indicating that certain regions of the biome may experience an increase in plant species richness under future climate change scenarios (Zwiener et al. 2017). Palm species richness, in particular, is strongly influenced by climatic factors, with water availability and precipitation emerging as the main determinants of this diversity (Bjorholm et al. 2005; Renninger and Phillips 2016). Consequently, the projected drier conditions for the future may exacerbate these dynamics, disproportionately impacting high‐richness areas and altering current distribution patterns of palm species.

Our results reveal contrasting responses of palm species to future climate change in the Atlantic Forest, with most species projected to lose suitable areas, while others show potential gains. These projections corroborate previous studies that also indicated substantial declines in climatically suitable areas for palms of the genus *Euterpe* under climate change (Marques et al. 2024). For example, *Euterpe edulis* was projected to lose suitable areas in our study, consistent with the pattern found by Sales et al. (2021) and Quitete Portela et al. (2023) when considering changes in vegetation cover. Such range declines in species already threatened by overexploitation may further compromise ecosystem services, generating cascading effects on trophic interactions, including frugivory and seed dispersal that sustain forest regeneration.

In contrast, some species are expected to expand their distributions under all future scenarios. It is important to note, however, that non‐climatic factors, such as biotic interactions and dispersal limitations, also influence species' responses, potentially constraining range shifts even when new areas become climatically suitable (Soberon 2007; Gaston 2009). As these processes were not incorporated into our ecological niche models, projected gains should therefore be interpreted as potential rather than guaranteed climate‐driven shifts. These divergent responses reflect species‐specific climatic sensitivities and the potential for biogeographic reorganization within the biome, with important implications for biodiversity and ecosystem services (Zwiener et al. 2017). Moreover, ongoing deforestation, habitat fragmentation, and land‐use change in the Atlantic Forest may exacerbate climate‐driven responses, further limiting species' ability to track suitable conditions. Conservation strategies must therefore integrate both climate and land‐use dynamics to be effective in safeguarding palms and associated ecosystem services.

Our results also revealed that species with more restricted distributions tend to reduce proportionally their future distribution areas, while species with broader distributions have the potential to expand their areas. This pattern likely reflects biologically meaningful differences in niche breadth and climatic tolerance among species, as widely distributed species tend to occupy a greater diversity of environmental conditions, being more generalist and, therefore, having a greater potential to benefit from these changes by colonizing newly suitable areas (Boulangeat et al. 2012). This interpretation is consistent with global‐scale evidence for plants demonstrating a strong positive relationship between range size and niche breadth, whereby species occupying more heterogeneous climatic conditions tend to exhibit broader environmental tolerances, reinforcing the importance of climatic heterogeneity in shaping geographic distributions and species' capacity to persist under climate change (Moulatlet et al. 2025). In contrast, species with more restricted distributions tend to reduce their future suitable areas, likely due to their specialization and narrower ecological niches, making them more vulnerable to climate change (Slatyer et al. 2013). This differential vulnerability has important implications for ecosystem dynamics, as the disproportionate loss of specialized species could alter key ecological interactions, such as seed dispersal and forest regeneration, under future climate scenarios.

It is important to point out that the models presented here showed low explanatory power, indicating that only 12%–22% of the variation is explained; therefore, it is important to interpret our results with caution. Notably, variables expected to influence climate responses, such as habitat type, stem height, and fruit size, were excluded from the best models. This suggests that our hypotheses (ii–iv) regarding trait‐mediated responses were not supported in studied scenarios. Although stem height and fruit size are known to mediate ecological strategies in plants, including competitive ability, survival under environmental stress, generation time, dispersal, and seedling establishment (Westoby 1998), these traits did not emerge as significant predictors of area change in our models. This may reflect the influence of other factors not included in our models, such as biotic interactions, dispersal limitations, and trait combinations (e.g., Andrew et al. 2022; Sales et al. 2021; Quitete Portela et al. 2023). Although palms exhibit considerable phenotypic diversity, the complexity of their life‐history strategies means that single traits may only partially capture species' responses to climate change, which was not evident in our data. For example, stem height may only become a limiting factor under extreme drought, surpassing physiological thresholds that make water transport less efficient and more prone to failure under water stress (Emilio et al. 2019; Tomlinson 2006), whereas fruit size influences dispersal success through interactions with frugivores, which were not modeled in our study. Similarly, habitat type did not emerge as a significant predictor of projected area change in our analysis. This may indicate that broad habitat categories do not sufficiently capture species‐specific climatic sensitivities or microclimatic buffering capacity. Climate change may therefore affect species across habitat types in comparable ways, with baseline range size playing a more decisive role than ecological traits. These mechanisms suggest that, while informative, traits such as stem height, habitat, and fruit size may not fully explain the observed variation in climate‐driven range shifts, highlighting the need for integrative approaches that combine traits, ecological interactions, and dispersal. While our results limit the ability to draw strong generalizations about trait‐based vulnerability, they highlight the complexity of predicting species' climate responses and emphasize the need for future research integrating traits, dispersal ecology, and biotic interactions to better understand the mechanisms underlying differential species sensitivity to climate change.

## 5 Conclusion

This study highlights regions within the Atlantic Forest with the highest potential for palm species loss and gain, with the greatest projected losses occurring along the northern coast and central areas of the biome. We estimate changes in climatically suitable areas, both in terms of losses and potential expansions, providing a broader view of the future of palms in the biome. For example, species such as *Syagrus lorenzoniorum, Attalea apoda, Acrocomia intumescens*, and *Attalea oleifera* were among the most negatively affected species under future climate scenarios, highlighting their heightened vulnerability to climate change. Our results also indicate that species with more restricted distributions are more likely to lose suitable areas in future climate change scenarios compared to species with wider distributions. Contrary to our initial expectations, habitat type, stem height, and fruit size examined here did not show consistent predictive power across scenarios. These findings have direct conservation implications. Prioritizing species with restricted distributions and regions projected to experience losses in species richness should be central to conservation planning. Actions may include identifying and protecting climatically stable refugia, implementing habitat restoration in areas most likely to remain suitable, and promoting landscape connectivity to facilitate the movement of palm‐dispersing species. By focusing conservation efforts on the most vulnerable species and regions, it becomes possible to more effectively maintain palm diversity and ecosystem resilience in the Atlantic Forest, one of the world's most threatened biodiversity hotspots.

## Author Contributions

**Júlia Angeli:** conceptualization (equal), data curation (lead), formal analysis (lead), methodology (lead), software (lead), validation (equal), writing – original draft (lead), writing – review and editing (lead). **Daniela Custódio Talora:** conceptualization (supporting), supervision (supporting), validation (supporting), writing – review and editing (supporting). **Gabriela Alves‐Ferreira:** formal analysis (supporting), methodology (supporting), software (supporting), validation (supporting), writing – review and editing (supporting). **Neander Marcel Heming:** formal analysis (supporting), methodology (supporting), software (supporting), validation (supporting). **Eliana Cazetta:** conceptualization (equal), formal analysis (supporting), methodology (supporting), supervision (lead), validation (supporting), writing – review and editing (supporting).

## Conflicts of Interest

The authors declare no conflicts of interest.

## Supporting information


**Table S1:** List of 83 palm species, including references, total number of cleaned occurrence records, and number of records within the Atlantic Forest.
**Table S2:** Trait data of 59 Atlantic Forest palms, including habitat, maximum stem height (m), maximum stem diameter (cm), average fruit length (cm), and average fruit width (cm). References are provided at the end of the table, according to the symbols.
**Table S3:** Selected climatic variables for each of the 59 palm species, based on the 19 bioclimatic variables derived from the WorldClim v2.1 platform (Fick and Hijmans 2017).
**Table S4:** Phylogenetic signal (Pagel's λ and Fritz & Purvis' D) for functional traits of Atlantic Forest palms. Continuous traits were evaluated using Pagel's λ and the binary trait (habitat) using D. * indicates significant *p*‐values.
**Table S5:** Model selection for the response variable: area change ratio under the optimistic 2050 scenario.
**Table S6:** Model selection for the response variable: area change ratio under the pessimistic 2050 scenario.
**Table S7:** Model selection for the response variable: area change ratio under the optimistic 2070 scenario.
**Table S8:** Model selection for the response variable: area change ratio under the pessimistic 2070 scenario.
**Table S9:** Ratio of area change for each of the 59 palm species under future climate scenarios relative to the current (baseline) scenario. A value of 0 indicates complete loss of the suitable area, 1 indicates no change, values greater than 1 indicate an expansion, and values between 0 and 1 indicate a contraction of the suitable area under future climate conditions.
**Figure S1:** Phylogenetic tree of the 59 palm species from the Atlantic Forest used in the PGLS analysis.
**Figure S2:** Graphs illustrate the percentage of the area lost (red) and the percentage of the area gained (blue) in each future climate change scenario, for each palm species.

## Data Availability

All data used in this study, along with the Supporting Information, are available in the Figshare Digital Repository: doi: https://doi.org/10.6084/m9.figshare.c.7768481.
