# Supplementary material for: Climate Change Vulnerability and Conservation Priorities for Atlantic Forest Palms
Source: Ecol Evol. 2026 Apr 8;16(4):e73411. doi: 10.1002/ece3.73411 (PMC13062646; doi:10.1002/ece3.73411)
Supplement: Supplementary file 1 — Table S1: List of 83 palm species, including references, total number of cleaned occurrence records, and number of records within the Atlantic Forest. Table S2: Trait data of 59 Atlantic Forest palms, including habitat, maximum stem height (m), maximum stem diameter (cm), average fruit length (cm), and average fruit width (cm). References are provided at the end of the table, according to the symbols. Table S3: Selected climatic variables for each of the 59 palm species, based on the 19 bioclimatic variables derived from the WorldClim v2.1 platform (Fick and Hijmans 2017). Table S4: Phylogenetic signal (Pagel's λ and Fritz & Purvis' D) for functional traits of Atlantic Forest palms. Continuous traits were evaluated using Pagel's λ and the binary trait (habitat) using D. * indicates significant p‐values. Table S5: Model selection for the response variable: area change ratio under the optimistic 2050 scenario. Table S6: Model selection for the response variable: area change ratio under the pessimistic 2050 scenario. Table S7: Model selection for the response variable: area change ratio under the optimistic 2070 scenario. Table S8: Model selection for the response variable: area change ratio under the pessimistic 2070 scenario. Table S9: Ratio of area change for each of the 59 palm species under future climate scenarios relative to the current (baseline) scenario. A value of 0 indicates complete loss of the suitable area, 1 indicates no change, values greater than 1 indicate an expansion, and values between 0 and 1 indicate a contraction of the suitable area under future climate conditions. Figure S1: Phylogenetic tree of the 59 palm species from the Atlantic Forest used in the PGLS analysis. Figure S2: Graphs illustrate the percentage of the area lost (red) and the percentage of the area gained (blue) in each future climate change scenario, for each palm species. [file ECE3-16-e73411-s001.docx]

# Supplementary Material

**Climate change vulnerability and conservation priorities for Atlantic Forest palms**

Júlia Angeli, Daniela Custódio Talora, Gabriela Alves-Ferreira, Neander Marcel Heming, Eliana Cazetta

# Table S1. List of 83 palm species, including references, total number of cleaned occurrence records, and number of records within the Atlantic Forest.

# * Indicates species without occurrence records in the Atlantic Forest after geographical bias correction, which were excluded from Ecological Niche Modeling, and ** Indicates species without the minimum of 5 occurrence records after geographical bias correction, which were excluded from Ecological Niche Modeling.

| **Species** | **Reference** | **All records** | **Atlantic Forest records** |
| --- | --- | --- | --- |
| *Acrocomia aculeata* | Cerqueira et al., 2023/ Bello et al., 2017 | 841 | 74 |
| *Acrocomia intumescens* | Cerqueira et al., 2023 | 35 | 20 |
| *Allagoptera arenaria* | Cerqueira et al., 2023/Bello et al., 2017 | 94 | 72 |
| *Allagoptera brevicalyx* | Cerqueira et al., 2023 | 23 | 17 |
| *Allagoptera caudescens* | Cerqueira et al., 2023 | 69 | 65 |
| *Astrocaryum aculeatissimum* | Cerqueira et al., 2023/Bello et al., 2017 | 91 | 88 |
| *Attalea apoda* | Cerqueira et al., 2023 | 5 | 5 |
| *Attalea burretiana* | Cerqueira et al., 2023 | 27 | 23 |
| *Attalea dubia* | Cerqueira et al., 2023/Bello et al., 2017 | 34 | 29 |
| *Attalea funifera* | Cerqueira et al., 2023 | 39 | 32 |
| *Attalea humilis* | Cerqueira et al., 2023 | 45 | 41 |
| *Attalea oleifera* | Cerqueira et al., 2023/Bello et al., 2017 | 28 | 15 |
| *Attalea seabrensis ** | Cerqueira et al., 2023 | 10 | 0 |
| *Attalea voeksii *** | Cerqueira et al., 2023 | 1 |  |
| *Bactris acanthocarpa* | Cerqueira et al., 2023/Bello et al., 2017 | 268 | 39 |
| *Bactris bahiensis* | Cerqueira et al., 2023 | 34 | 33 |
| *Bactris caryotifolia* | Cerqueira et al., 2023 | 32 | 30 |
| *Bactris ferruginea* | Cerqueira et al., 2023 | 50 | 48 |
| *Bactris gasipaes* | Bello et al., 2017 | 551 | 17 |
| *Bactris glassmanii* | Cerqueira et al., 2023 | 29 | 23 |
| *Bactris hatschbachii* | Cerqueira et al., 2023 | 12 | 11 |
| *Bactris hirta* | Cerqueira et al., 2023 | 280 | 31 |
| *Bactris horridispatha* | Cerqueira et al., 2023 | 14 | 11 |
| *Bactris pickelli* | Cerqueira et al., 2023 | 31 | 23 |
| *Bactris setosa* | Cerqueira et al., 2023/Bello et al., 2017 | 160 | 136 |
| *Bactris soeiroana *** | Cerqueira et al., 2023 | 3 |  |
| *Bactris timbuiensis *** | Cerqueira et al., 2023 | 4 |  |
| *Bactris vulgaris* | Cerqueira et al., 2023 | 59 | 57 |
| *Butia capitata* | Bello et al., 2017 | 160 | 20 |
| *Butia catarinensis* | Cerqueira et al., 2023 | 19 | 17 |
| *Butia eriospatha* | Cerqueira et al., 2023 | 59 | 47 |
| *Butia exilata *** | Cerqueira et al., 2023 | 4 |  |
| *Butia microspadix* | Cerqueira et al., 2023 | 12 | 5 |
| *Butia odorata* | Cerqueira et al., 2023 | 201 | 21 |
| *Butia paraguayensis* | Cerqueira et al., 2023 | 95 | 10 |
| *Butia pubispatha *** | Cerqueira et al., 2023 | 1 |  |
| *Butia yatay* | Cerqueira et al., 2023 | 63 | 3 |
| *Desmoncus orthacanthos* | Cerqueira et al., 2023 | 238 | 67 |
| *Desmoncus polyacanthos* | Cerqueira et al., 2023 | 423 | 74 |
| *Euterpe edulis* | Cerqueira et al., 2023/Bello et al., 2017 | 446 | 351 |
| *Geonoma bondariana *** | Cerqueira et al., 2023 | 2 |  |
| *Geonoma brevispatha* | Cerqueira et al., 2023 | 107 | 36 |
| *Geonoma conduruenses *** | Cerqueira et al., 2023 | 2 |  |
| *Geonoma elegans* | Cerqueira et al., 2023/Bello et al., 2017 | 110 | 100 |
| *Geonoma fiscellaria* | Cerqueira et al., 2023 | 6 | 6 |
| *Geonoma gamiova* | Cerqueira et al., 2023/Bello et al., 2017 | 82 | 80 |
| *Geonoma gastoniana *** | Cerqueira et al., 2023 | 3 |  |
| *Geonoma littoralis *** | Cerqueira et al., 2023 | 1 |  |
| *Geonoma kuhlmannii *** | Cerqueira et al., 2023 | 4 |  |
| *Geonoma pauciflora* | Cerqueira et al., 2023/Bello et al., 2017 | 68 | 57 |
| *Geonoma pohliana* | Cerqueira et al., 2023 | 129 | 108 |
| *Geonoma rodeiensis* | Cerqueira et al., 2023 | 10 | 10 |
| *Geonoma rubescens* | Cerqueira et al., 2023 | 16 | 15 |
| *Geonoma schottiana* | Cerqueira et al., 2023/Bello et al., 2017 | 232 | 209 |
| *Geonoma trinervis *** | Cerqueira et al., 2023 | 0 |  |
| *Geonoma wittigiana* | Cerqueira et al., 2023 | 7 | 7 |
| *Roystonea oleraceae* | Bello et al., 2017 | 106 | 27 |
| *Syagrus amicorum *** | Cerqueira et al., 2023 | 2 |  |
| *Syagrus ×andrequiceana *** | Cerqueira et al., 2023 | 1 |  |
| *Syagrus botryophora* | Cerqueira et al., 2023 | 33 | 28 |
| *Syagrus×camposportoana *** | Cerqueira et al., 2023 | 1 |  |
| *Syagrus cearensis* | Cerqueira et al., 2023 | 51 | 12 |
| *Syagrus ×cipoensis *** | Cerqueira et al., 2023 | 1 |  |
| *Syagrus guaratinguensis *** | Cerqueira et al., 2023 | 0 |  |
| *Syagrus hoehnei* | Cerqueira et al., 2023 | 11 | 10 |
| *Syagrus insignis* | Cerqueira et al., 2023 | 13 | 13 |
| *Syagrus itapebiensis *** | Cerqueira et al., 2023 | 1 |  |
| *Syagrus kellyana *** | Cerqueira et al., 2023 | 1 |  |
| *Syagrus ×lacerdamourae *** | Cerqueira et al., 2023 | 0 |  |
| *Syagrus lorenzoniorum* | Cerqueira et al., 2023 | 6 | 6 |
| *Syagrus macrocarpa* | Cerqueira et al., 2023 | 11 | 9 |
| *Syagrus oleracea* | Bello et al., 2017 | 84 | 22 |
| *Syagrus picrophylla* | Cerqueira et al., 2023 | 13 | 10 |
| *Syagrus pseudococos* | Cerqueira et al., 2023/Bello et al., 2017 | 28 | 25 |
| *Syagrus romanzifollia *** | Bello et al., 2017 | 0 |  |
| *Syagrus romanzoffiana* | Cerqueira et al., 2023/Bello et al., 2017 | 1372 | 289 |
| *Syagrus ruschiana* | Cerqueira et al., 2023/Bello et al., 2017 | 17 | 17 |
| *Syagrus santosii *** | Cerqueira et al., 2023 | 1 |  |
| *Syagrus schizophylla* | Cerqueira et al., 2023 | 72 | 43 |
| *Syagrus ×serroana *** | Cerqueira et al., 2023 | 1 |  |
| *Syagrus ×tostana *** | Cerqueira et al., 2023 | 1 |  |
| *Syagrus weddelliana* | Cerqueira et al., 2023 | 15 | 9 |
| *Trithrinax acanthocoma* | Cerqueira et al., 2023 | 8 | 5 |

# Table S2. Trait data of 59 Atlantic Forest palms, including habitat, maximum stem height (m), maximum stem diameter (cm), average fruit length (cm), and average fruit width (cm). References are provided at the end of the table, according to the symbols.

| **Species** | **Habitat** | **Maximum Stem Height (m)** | **Maximum Stem Diameter (cm)** | **Average Fruit**  **Length (cm)** | **Average**  **Fruit Width (cm)** |
| --- | --- | --- | --- | --- | --- |
| *Acrocomia aculeata ▪* | Open-area | 12 | 50 | 4.25 | 4.6 |
| *Acrocomia intumescens ▪* | Forest-interior | 8 ᶧ | 50 ˘ ˘ | 4.6 | 4.6 |
| *Allagoptera arenaria ▪* | Open-area | 0.2 | 15 | 1.6 | 1.15 |
| *Allagoptera brevicalyx ▪* | Open-area | 2 | 20 ˘ ˘ | 1.75 | 1.25 |
| *Allagoptera caudescens ▪* | Open-area | 8 | 25 | 4.25 | 3.25 |
| *Astrocaryum aculeatissimum ▪* | Open-area | 8 | 15 | 5.5 | 3.5 |
| *Attalea apoda ▪* | Open-area | 13 | 36.7 ˘ ˘ | 6 | 3.3 |
| *Attalea burretiana ▪* | Forest-interior | 30 ᶧ | 40 ᶧ ᶧ | 7.27 | 4.55 |
| *Attalea dubia ▪* | Open-area | 25 | 35 | 6.25 | 3 |
| *Attalea funifera ▪* | Open-area | 15 | 30 | 12.5 | 6 |
| *Attalea humilis ▪* | Open-area | 1 ᶧ | - | 6.5 | 5.25 |
| *Attalea oleifera ▪* | Open-area | 30 | 45 | 9 | 5 |
| *Bactris acanthocarpa ▪* | Forest-interior | 2 | 7 | 1.4 | 1.4 |
| *Bactris bahiensis ▪* | Open-area | 3 | 1 | 1.5 | 1.1 |
| *Bactris caryotifolia ▪* | Forest-interior | 1.5 | 2 | 1.4 | 1.4 |
| *Bactris ferruginea ▪* | Open-area | 10 | 10 | 1.65 | 1.65 |
| *Bactris gasipaes ▪* | Open-area | 18 | 25 | 5 | 4 |
| *Bactris glassmanii ▪* | Open-area | 3 | 2 | 1.3 | 1.3 |
| *Bactris hatschbachii ▪* | Forest-interior | 5 | 2.5 | 2.05 | 2 |
| *Bactris hirta ▪* | Open-area | 3 | 2 | 0.75 | 0.75 |
| *Bactris horridispatha ▪* | Forest-interior | 6 | 3 | 3.25 | 2.4 |
| *Bactris pickelii ▪* | Forest-interior | 3 | 1.5 | 1.35 | 1.35 |
| *Bactris setosa ▪* | Forest-interior | 6 | 4 | 1.25 | 1.75 |
| *Bactris vulgaris ▪* | Open-area | 3 | 3.5 | 1.7 | 1.7 |
| *Butia capitata ▪* | Open-area | 6 | 50 | 2.65 | 1.7 |
| *Butia catarinensis ▪* | Open-area | 0.8 ᶧ | 30 ᶧ | 2.5 | 1.2 |
| *Butia eriospatha ▪* | Open-area | 6 | 50 | 1.9 | 1.75 |
| *Butia microspadix ▪* | Open-area | - | - | 2 | 1.05 |
| *Butia odorata ▪* | Open-area | 9 ᶧ | 30 ᶧ | 2.2 | 1.85 |
| *Butia paraguayensis ▪* | Open-area | 4 | 20 | 2.95 | 1.85 |
| *Butia yatay ▪* | Open-area | 12 | 40 | 3.6 | 2.65 |
| *Desmoncus orthacanthos ▪* | Open-area | 20 | 3 | 1.49 | 1.42 |
| *Desmoncus polyacanthos ▪* | Forest-interior | 37 | 3.5 | 2 | 1 |
| *Euterpe edulis ▪* | Forest-interior | 12 | 15 | 1.2 | 1.2 |
| *Geonoma brevispatha ᶧ* | Forest-interior | 3 | 4 | 0.95 | 0.7 |
| *Geonoma elegans ▪* | Forest-interior | 3 | 1 | 0.95 | 0.72 |
| *Geonoma fiscellaria ᶧ* | Forest-interior | 4.5 | 4 | 1.6 | 1.4 |
| *Geonoma gamiova ⁼* | Forest-interior | 3.4 | 3 | 1.225 | 0.95 |
| *Geonoma pauciflora ▪* | Forest-interior | 3 | 1.5 | 0.96 | 0.78 |
| *Geonoma pohliana ▪* | Forest-interior | 7.5 | 7 | 1 | 0.735 |
| *Geonoma rodeiensis ▪▪* | Forest-interior | 3 | 3.15 ˘ ˘ | 1 | 0.84 ˘ ˘ |
| *Geonoma rubescens ᶧ* | Forest-interior | 2.2 | 2.5 | 1 | 0.9 |
| *Geonoma schottiana ▪* | Forest-interior | 4 | 4 | 0.93 | 0.825 |
| *Geonoma wittigiana ᶧ* | Forest-interior | 2.2 | 1.4 | 0.6 ⁼ ⁼ | 0.55 |
| *Roystonea oleracea ▪* | Open-area | 40 | 66 | 1.5 | 0.9 |
| *Syagrus botryophora ▪* | Open-area | 18 | 25 | 4.25 | 2.6 |
| *Syagrus cearensis ▪* | Open-area | 10 | 18 | 3.75 | 3.5 |
| *Syagrus hoehnei ͌* | Forest-interior | 5 | 15 | 3.15 | 2.6 |
| *Syagrus insignis ͌ ͌* | Forest-interior | 11 | 10 | 2.05 | 1.8 |
| *Syagrus lorenzoniorum ▪* | Forest-interior | 4 | 16 | 2.75 | 2.5 |
| *Syagrus macrocarpa ▪* | Forest-interior | 10 | 20 | 7.75 | 3.75 |
| *Syagrus oleracea ▪* | Open-area | 20 | 30 | 5.5 | 2.35 |
| *Syagrus picrophylla ▪* | Forest-interior | 8 | 20 | 3.5 | 2.3 |
| *Syagrus pseudococos ▪* | Forest-interior | 15 | 25 | 6.5 | 3.9 |
| *Syagrus romanzoffiana ▪* | Open-area | 17 | 50 | 2.5 | 1.5 |
| *Syagrus ruschiana ▪* | Open-area | 8 | 12 | 2.5 | 2 |
| *Syagrus schizophylla ▪* | Open-area | 4 | 15 | 2.75 | 2 |
| *Syagrus weddelliana ˘* | Forest-interior | 3 | 10 | 1.85 | 1.75 |
| *Trithrinax acanthocoma ᶧ* | Open-area | 15 | 35 | 0.94 ˘ ˘ | 1.85 |

▪ Kissling, W. D. et al. PalmTraits 1.0, a species-level functional trait database of palms worldwide. Scientific Data, v. 6, n. 1, p. 178, 24 set. 2019.

▪▪ Lima, Amélia Lopes; Soares, João Juares. Aspectos florísticos e ecológicos de palmeiras (Arecaceae) da Reserva Biológica de Duas Bocas, Cariacica, Espírito Santo. CEP, v. 29060, p. 900, 2003.

ᶧ Lorenzi, H., Noblick, L., Kahn, F., Ferreira, E.J.L., 2010. Flora Brasileira: Arecaceae (Palmeiras). Instituto Plantarum, Nova Odessa, SP.

ᶧ ᶧ Soares, K.P. Attalea in Flora e Funga do Brasil. Jardim Botânico do Rio de Janeiro. Available at: <https://floradobrasil.jbrj.gov.br/FB43612>. Accessed on April 29, 2024.

⁼ Soares, K.P.; Leitman, P.M. Geonoma in Flora e Funga do Brasil. Jardim Botânico do Rio de Janeiro. Available at: <https://floradobrasil.jbrj.gov.br/FB34045>. Accessed on April 29, 2024.

⁼ ⁼ Soares, K.P.; Leitman, P.M. Geonoma in Flora e Funga do Brasil. Jardim Botânico do Rio de Janeiro. Available at: <https://floradobrasil.jbrj.gov.br/FB44422>. Accessed on April 29, 2024.

͌ Soares, K.P. Syagrus in Flora e Funga do Brasil. Jardim Botânico do Rio de Janeiro. Available at: <https://floradobrasil.jbrj.gov.br/FB44820>. Accessed on April 29, 2024.

͌ ͌ Soares, K.P. Syagrus in Flora e Funga do Brasil. Jardim Botânico do Rio de Janeiro. Available at: <https://floradobrasil.jbrj.gov.br/FB582739>. Accessed on April 29, 2024.

˘ Soares, K.P. Syagrus in Flora e Funga do Brasil. Jardim Botânico do Rio de Janeiro. Available at: <https://floradobrasil.jbrj.gov.br/FB44834>. Accessed on April 29, 2024.

˘ ˘ Genus average

# Table S3. Selected climatic variables for each of the 59 palm species, based on the 19 bioclimatic variables derived from the WorldClim v2.1 platform (Fick and Hijmans, 2017).

| **Species** | **Selected bioclimatic variables** |
| --- | --- |
| *Acrocomia aculeata* | Bio 13/ Bio 14/ Bio 15/ Bio 18/ Bio 19/ Bio 2/ Bio 8/ Bio 9 |
| *Acrocomia intumescens* | Bio 16/ Bio 19/ Bio 4/ Bio 7/ Bio 8 |
| *Allagoptera arenaria* | Bio 1/ Bio 10/ Bio 13/ Bio 14/ Bio 15/ Bio 18/ Bio 19/ Bio 2/ Bio 8/ Bio 9 |
| *Allagoptera brevicalyx* | Bio 13/ Bio 19/ Bio 4/ Bio 8 |
| *Allagoptera caudescens* | Bio 12/ Bio 13/ Bio 14/ Bio 3/ Bio 5 |
| *Astrocaryum aculeatissimum* | Bio 15/ Bio 16/ Bio 2/ Bio 3/ Bio 4/ Bio 8 |
| *Attalea apoda* | Bio 13/ Bio 15/ Bio 2/ Bio 3 |
| *Attalea burretiana* | Bio 13/ Bio 15/ Bio 18/ Bio 3/ Bio 4/ Bio 7/ Bio 8 |
| *Attalea dubia* | Bio 13/ Bio 15/ Bio 18/ Bio 19/ Bio 2/ Bio 3/ Bio 8 |
| *Attalea funifera* | Bio 10/ Bio 16/ Bio 18/ Bio 19/ Bio 4/ Bio 7/ Bio 8 |
| *Attalea humilis* | Bio 12/ Bio 19/ Bio 3/ Bio 5 |
| *Attalea oleifera* | Bio 12/ Bio 14/ Bio 18/ Bio 3/ Bio 4/ Bio 8 |
| *Bactris acanthocarpa* | Bio 13/ Bio 15/ Bio 18/ Bio 19/ Bio 2/ Bio 3/ Bio 4/ Bio 5/ Bio 8 |
| *Bactris bahiensis* | Bio 12/ Bio 14/ Bio 3/ Bio 5 |
| *Bactris caryotifolia* | Bio 12/ Bio 18/ Bio 19/ Bio 3/ Bio 5 |
| *Bactris ferruginea* | Bio 12/ Bio 14/ Bio 18/ Bio 19/ Bio 3/ Bio 8 |
| *Bactris gasipaes* | Bio 10/ Bio 13/ Bio 14/ Bio 15/ Bio 18/ Bio 19/ Bio 2/ Bio 3/ Bio 8/ Bio 9 |
| *Bactris glassmanii* | Bio 17/ Bio 19/ Bio 4/ Bio 8 |
| *Bactris hatschbachii* | Bio 12/ Bio 17/ Bio 3/ Bio 4/ Bio 5 |
| *Bactris hirta* | Bio 13/ Bio 15/ Bio 18/ Bio 19/ Bio 2/ Bio 3/ Bio 4/ Bio 5/ Bio 8 |
| *Bactris horridispatha* | Bio 12/ Bio 15/ Bio 16/ Bio 18/ Bio 3/ Bio 4/ Bio 8 |
| *Bactris pickelli* | Bio 10/ Bio 19/ Bio 2/ Bio 3/ Bio 4/ Bio 8 |
| *Bactris setosa* | Bio 12/ Bio 15/ Bio 18/ Bio 19/ Bio 2/ Bio 3/ Bio 8 |
| *Bactris vulgaris* | Bio 11/ Bio 13/ Bio 15/ Bio 19/ Bio 3/ Bio 8/ Bio 9 |
| *Butia capitata* | Bio 10/ Bio 13/ Bio 14/ Bio 15/ Bio 18/ Bio 19/ Bio 2/ Bio 3/ Bio 8 |
| *Butia catarinensis* | Bio 12/ Bio 13/ Bio 18/ Bio 2/ Bio 5/ Bio 7/ Bio 9 |
| *Butia eriospatha* | Bio 10/ Bio 13/ Bio 14/ Bio 15/ Bio 18/ Bio 19/ Bio 2/ Bio 3/ Bio 8/ Bio 9 |
| *Butia microspadix* | Bio 12/ Bio 18/ Bio 2/ Bio 4/ Bio 7/ Bio 8 |
| *Butia odorata* | Bio 13/ Bio 14/ Bio 15/ Bio 18/ Bio 19/ Bio 2/ Bio 3/ Bio 8/ Bio 9 |
| *Butia paraguayensis* | Bio 12/ Bio 13/ Bio 18/ Bio 2/ Bio 5/ Bio 7/ Bio 8/ Bio 9 |
| *Butia yatay* | Bio 1 /Bio 10 /Bio 13 /Bio 14 /Bio 15 /Bio 18 /Bio 19 /Bio 2 /Bio 8 /Bio 9 |
| *Desmoncus orthacanthos* | Bio 13 /Bio 15 /Bio 18 /Bio 19 /Bio 2 /Bio 4 /Bio 8 |
| *Desmoncus polyacanthos* | Bio 13/ Bio 15/ Bio 18/ Bio 19/ Bio 2/ Bio 4/ Bio 5 |
| *Euterpe edulis* | Bio 13/ Bio 14/ Bio 15/ Bio 18/ Bio 19/ Bio 2/ Bio 3/ Bio 8/ Bio 9 |
| *Geonoma brevispatha* | Bio 13/ Bio 15/ Bio 18/ Bio 19/ Bio 2/ Bio 3/ Bio 8 |
| *Geonoma elegans* | Bio 10/ Bio 12/ Bio 18/ Bio 19/ Bio 2/ Bio 3/ Bio 7 |
| *Geonoma fiscellaria* | Bio 13/ Bio 15/ Bio 2/ Bio 3/ Bio 4/ Bio 5 |
| *Geonoma gamiova* | Bio 12/ Bio 16/ Bio 19/ Bio 2/ Bio 5/ Bio 7/ Bio 8 |
| *Geonoma pauciflora* | Bio 13/ Bio 15/ Bio 18/ Bio 19/ Bio 2/ Bio 3/ Bio 4/ Bio 5 |
| *Geonoma pohliana* | Bio 13/ Bio 15/ Bio 18/ Bio 19/ Bio 3/ Bio 7/ Bio 8 |
| *Geonoma rodeiensis* | Bio 12/ Bio 14/ Bio 3/ Bio 5 |
| *Geonoma rubescens* | Bio 11/ Bio 15/ Bio 16/ Bio 3/ Bio 6/ Bio 9 |
| *Geonoma schottiana* | Bio 12/ Bio 15/ Bio 16/ Bio 18/ Bio 2/ Bio 7/ Bio 8 |
| *Geonoma wittigiana* | Bio 13/ Bio 15/ Bio 3/ Bio 4/ Bio 8 |
| *Roystonea oleraceae* | Bio 1/ Bio 13/ Bio 14/ Bio 15/ Bio 18/ Bio 19/ Bio 2/ Bio 3 |
| *Syagrus botryophora* | Bio 17/ Bio 18/ Bio 19/ Bio 2/ Bio 5/ Bio 8 |
| *Syagrus cearensis* | Bio 14/ Bio 16/ Bio 18/ Bio 19/ Bio 2/ Bio 4/ Bio 8 |
| *Syagrus hoehnei* | Bio 13/ Bio 17/ Bio 3/ Bio 4/ Bio 9 |
| *Syagrus insignis* | Bio 13/ Bio 2/ Bio 3 |
| *Syagrus lorenzoniorum* | Bio 12/ Bio 14/ Bio 5 |
| *Syagrus macrocarpa* | Bio 10/ Bio 13/ Bio 15/ Bio 18/ Bio 19/ Bio 3/ Bio 4/ Bio 7 |
| *Syagrus oleracea* | Bio 13/ Bio 14/ Bio 15/ Bio 18/ Bio 19/ Bio 2/ Bio 4/ Bio 5 |
| *Syagrus picrophylla* | Bio 17/ Bio 18/ Bio 19/ Bio 2/ Bio 5/ Bio 7/ Bio 8 |
| *Syagrus pseudococos* | Bio 12/ Bio 19/ Bio 2/ Bio 3/ Bio 4/ Bio 5 |
| *Syagrus romanzoffiana* | Bio 10/ Bio 13/ Bio 14/ Bio 15/ Bio 18/ Bio 19/ Bio 2/ Bio 4 |
| *Syagrus ruschiana* | Bio 16/ Bio 4/ Bio 7/ Bio 8 |
| *Syagrus schizophylla* | Bio 10/ Bio 13/ Bio 14/ Bio 15/ Bio 18/ Bio 19/ Bio 2/ Bio 8/ Bio 9 |
| *Syagrus weddelliana* | Bio 14/ Bio 18/ Bio 19/ Bio 2/ Bio 3/ Bio 5/ Bio 8 |
| *Trithrinax acanthocoma* | Bio 14/ Bio 18/ Bio 19/ Bio 2/ Bio 8 |

ODMAP Protocol

## Overview

#### Authorship

Contact : [juliaangeli34@gmail.com](mailto:juliaangeli34@gmail.com)

Study link: In review

#### Model objective

Model objective: Forecast and transfer

Target output: Suitable vs. unsuitable habitat

#### Focal Taxon

Focal Taxon: Arecaceae

#### Location

Location: Brazilian Atlantic Forest

#### Scale of Analysis

Spatial extent: -57.39, -34.79, -33.75, -3.23 (xmin, xmax, ymin, ymax)

Spatial resolution: ~2.5 arc-min (~4.5 km²)

Temporal extent: We used occurrence records collected between 1970 and 2024

Temporal resolution: We used the present (1970–2000) and two future climate scenarios: 2050 (2041–2060) and 2070 (2061–2080). For each future scenario, we considered two Shared Socioeconomic Pathways (SSPs) representing optimistic (SSP245) and pessimistic (SSP585) greenhouse gas scenarios.

Boundary: natural

#### Biodiversity data

Observation type: We gathered occurrence records from the Global Biodiversity Information Facility (GBIF; www.gbif.org), SpeciesLink (<https://www.specieslink.net>), Integrated Digitized Biocollections (iDigBio; <https://www.idigbio.org>), and iNaturalist (<https://www.inaturalist.org>).

Response data type: presence-only

#### Predictors

Predictor types: Bioclimatic

#### Hypotheses

Hypotheses: Variation in the response of Atlantic Forest palms to climate change is partly explained by their functional traits. Specifically, we expect that species with restricted geographic ranges and forest-interior habits will experience greater reductions in climatically suitable area, whereas species with shorter stem height and larger fruits will have greater potential for range expansion. To evaluate this hypothesis, we used ecological niche models to project climatically suitable areas for the present and for future scenarios (2050 and 2070; SSP370 and SSP585) and related the projected changes in suitable area to the species’ functional traits.

#### Assumptions

Model assumptions: • Relevant ecological drivers of palm species distributions at a large scale are included. • Occurrence records are accurate, georeferenced correctly, and representative of each species’ distribution. • Species are assumed to be in equilibrium with their current environment. • Sampling effort is adequate and representative of the study area. • The niche is preserved over time.

#### Algorithms

Modelling techniques: maxent

Model complexity: The models were fitted and evaluated using the R package ENMwizard package (Heming et al., 2019). MaxEnt requires only occurrence data and bioclimatic variables (Phillips et al., 2006) and is widely recognized for its high performance and accuracy, even when applied to small samples (Hernandez et al., 2006). In addition, previous studies focusing on palms have also achieved good performance using MaxEnt (Blach-Overgaard et al., 2009; Blach-Overgaard et al., 2010). The models were fitted using options for Feature classes (FC) and Regularization multipliers (RMs), including all combinations of the following classes: linear (L), product (P), and quadratic (Q), along with RM values (ranging from 0.5 to 5, with increments of 0.5), resulting in 70 models per species

Model averaging: Only a single algorithm (MaxEnt) was used

#### Workflow

Model workflow: Model fitting We modeled the ecological niches of 59 Atlantic Forest palm species using the MaxEnt algorithm (v. 3.4.1) implemented through the ENMwizard package. Models were calibrated using occurrence records and bioclimatic variables. For each species, all combinations of linear (L), product (P), and quadratic (Q) feature classes and regularization multiplier (RM) values from 0.5 to 5 (increments of 0.5) were tested, resulting in 70 candidate models per species. Calibration areas were defined as a 1.5° buffer around the minimum convex polygon (MCP) of all occurrences. Model assessment Predictive performance was assessed using spatially independent partitioning methods: block partitioning for species with ≥15 occurrences, and jackknife partitioning for species with <15 occurrences. Models were evaluated based on omission rate (OR) and area under the curve (AUC), selecting the top 10% of models to construct a consensus per species. Prediction continuous predictions of climatic suitability were converted into binary maps (suitable/unsuitable) using a threshold that excluded 10% of occurrence points with the lowest suitability. These models were projected into future climate scenarios (2050 and 2070; SSP370 and SSP585). To reduce overprediction, we refined binary maps by intersecting them with the MCP of filtered occurrence points. Buffers were not applied due to the limited dispersal capacity of palms.

#### Software

Software: Models were developed using R (version 4.3.2) with the ENMwizard package (Heming et al., 2019) and ENMeval package (Muscarella et al., 2014). Ecological niche models were implemented using MaxEnt (version 3.4.1; Phillips et al., 2017).

Code availability: The data are available as a supplementary file accompanying the article.

Data availability: The data are available as a supplementary file accompanying the article.

## Data

#### Biodiversity data

Taxon names: Species: *Acrocomia aculeata, Acrocomia intumescens, Allagoptera arenaria, Allagoptera brevicalyx, Allagoptera caudescens, Astrocaryum aculeatissimum, Attalea apoda, Attalea burretiana, Attalea dubia, Attalea funifera, Attalea humilis, Attalea oleifera, Bactris acanthocarpa, Bactris bahiensis, Bactris caryotifolia, Bactris ferruginea, Bactris gasipaes, Bactris glassmanii, Bactris hatschbachii, Bactris hirta, Bactris horridispatha, Bactris pickelli, Bactris setosa, Bactris vulgaris, Butia capitata, Butia catarinensis, Butia eriospatha, Butia microspadix, Butia odorata, Butia paraguayensis, Butia yatay, Desmoncus orthacanthos, Desmoncus polyacanthos, Euterpe edulis, Geonoma brevispatha, Geonoma elegans, Geonoma fiscellaria, Geonoma gamiova, Geonoma pauciflora, Geonoma pohliana, Geonoma rodeiensis, Geonoma rubescens, Geonoma schottiana, Geonoma wittigiana, Roystonea oleraceae, Syagrus botryophora, Syagrus cearensis, Syagrus hoehnei, Syagrus insignis, Syagrus lorenzoniorum, Syagrus macrocarpa, Syagrus oleracea, Syagrus picrophylla, Syagrus pseudococos, Syagrus romanzoffiana, Syagrus ruschiana, Syagrus schizophylla, Syagrus weddelliana, Trithrinax acanthocoma.*

Taxonomic reference system: The nomenclature and synonyms used follow the Flora Brasileira: Arecaceae (Palmeiras) (Lorenzi et al., 2010) and Cerqueira et al. (2023)

Ecological level: species

Data sources: We compiled occurrence records from the online databases Global Biodiversity Information Facility (GBIF; www.gbif.org), SpeciesLink (<https://www.specieslink.net>), Integrated Digitized Biocollections (iDigBio; <https://www.idigbio.org>), and iNaturalist (<https://www.inaturalist.org>).

Sampling design: Occurrence records were spatially thinned to reduce spatial autocorrelation, removing points less than 10 km apart using the spThin algorithm. Records with inaccurate or incomplete coordinates, as well as those corresponding to municipality centroids, were removed. Only species with at least five occurrence records were included. The final dataset includes 63 palm species from the Atlantic Forest.

Sample size: The cleaned dataset comprises 2,708 occurrence records across 59 species of palms.

Cleaning: We cleaned the coordinates by removing inaccurate records, including those with incomplete coordinates and those corresponding to municipality centroids.

Absence data: No true absence data were available; models were trained using presence-only data.

Background data: Background points were drawn from the calibration area defined as a 1.5° buffer around the minimum convex polygon (MCP) of all occurrence points for each species.

#### Data partitioning

Validation data: Occurrence records were partitioned spatially using block partitioning for species with ≥15 records and jackknife partitioning for species with <15 records. In the block method, points were divided into four spatially independent blocks; in each iteration, three blocks were used for training and one for validation. In jackknife partitioning, one point was removed at a time for model evaluation.

Test data: Validation was performed using the held-out block or the removed point in jackknife partitioning. Model performance was assessed with AUC and omission rate (OR) metrics.

#### Predictor variables

Predictor variables: We used 19 bioclimatic variables from WorldClim v2.1. Variables exhibiting Pearson correlation >0.75 were removed to reduce collinearity; only uncorrelated variables were included per species.

Data sources: WorldClim v2.1 (<https://www.worldclim.org/>)

Spatial extent: -57.39, -34.79, -33.75, -3.23 (xmin, xmax, ymin, ymax)

Spatial resolution: ~2.5 arc-min (~4.5 km²)

Coordinate reference system: The coordinate reference system is WGS84 (EPSG:4326).

Temporal extent: Baseline climate: 1970–2000

#### Transfer data

Data sources: WorldClim v2.1 (<https://www.worldclim.org/>) future projections from three Global Circulation Models (GCMs): IPSL-CM6A-LR, MIROC6 and MRI-ESM2-0.

Spatial extent: -57.39, -34.79, -33.75, -3.23 (xmin, xmax, ymin, ymax)

Spatial resolution: ~2.5 arc-min (~4.5 km²)

Temporal extent: Future periods: 2050 (2041–2060) and 2070 (2061–2080)

Models and scenarios: We considered two Shared Socioeconomic Pathways (SSPs) representing optimistic (SSP245) and pessimistic (SSP585) greenhouse gas scenarios. We used Global Circulation Models (GCM) IPSL-CM6A-LR, MIROC6 and MRI-ESM2-0.

## Model

#### Multicollinearity

Multicollinearity: Pearson correlations were calculated among the 19 WorldClim v2.1 bioclimatic variables. Variables with correlation > 0.75 were removed, and only uncorrelated variables were retained for each species.

#### Model settings

maxent: featureRule (L, P, Q, L + P, L + Q, P + Q, L + P + Q), regularizationMultiplierSet (0.5, 1, 1.5, 2, 2.5, 3, 3.5, 4, 4.5. 5)

Model settings (extrapolation): Extrapolation was limited by clipping final binary outputs to the minimum convex polygon of filtered occurrences, ensuring predictions remained within the known environmental space of palm records.

#### Model estimates

Coefficients: We accessed the model performance by calculating the Area Under Curve (AUC) and Omission Rate (OR)

#### Analysis and Correction of non-independence

Spatial autocorrelation: We used spatial block cross-validation to minimize spatial autocorrelation between calibration and validation occurrence records.

#### Threshold selection

Threshold selection: Final continuous suitability maps (present and future) were converted to binary maps (1= climatically suitable, 0= unsuitable areas) using the threshold that helps minimize uncertainties associated with datasets compiled from aggregated sources (10-percentile training presence).

## Assessment

#### Performance statistics

Performance on training data: AUC, OR

Performance on validation data: AUC, OR

Performance on test data: Not applicable; no independent external test dataset was available.

#### Plausibility check

Response shapes: Response curves were visually inspected for ecological plausibility within MaxEnt outputs.

Expert judgement: Final maps were reviewed to ensure consistency with known species distributions.

## Prediction

#### Prediction output

Prediction unit: Final continuous models (present and future) and suitable areas vs. unsuitable areas maps (suitable areas = 1 and unsuitable areas = 0)

Post-processing: We clipped the models by the extent of the Brazilian Atlantic Forest.

#### Uncertainty quantification

Scenario uncertainty: Future projections incorporated three GCMs (IPSL-CM6A-LR, MIROC6 and MRI-ESM2-0) and two SSPs (245 and 585) for 2050 and 2070 to capture climate-model and scenario uncertainty.

Novel environments: To limit overprediction and projections into novel conditions, final binary outputs were clipped to the minimum convex polygon of filtered occurrence records. No buffers were added, reflecting the limited dispersal capacity of palms.

# Table S4. Phylogenetic signal (Pagel’s λ and Fritz & Purvis’ D) for functional traits of Atlantic Forest palms. Continuous traits were evaluated using Pagel’s λ and the binary trait (habitat) using D. * indicates significant p-values.

| **Trait** | **Type** | **Metric** | **Phylogenetic signal** | **P-value** | **Phylogenetic Pattern** |
| --- | --- | --- | --- | --- | --- |
| Average Fruit Length | Continuous | λ | 0.893921 | <0.001 | Strong |
| Average Fruit Width | Continuous | λ | 0.925224 | <0.001 | Strong |
| Max Stem Height | Continuous | λ | 0.60125 | 0.17361 | Moderate/Not significant |
| Max Stem Diameter | Continuous | λ | 0.942674 | <0.001 | Strong |
| Habitat | Binary | D | 0.4604238 | 0.001 | Moderate |

**
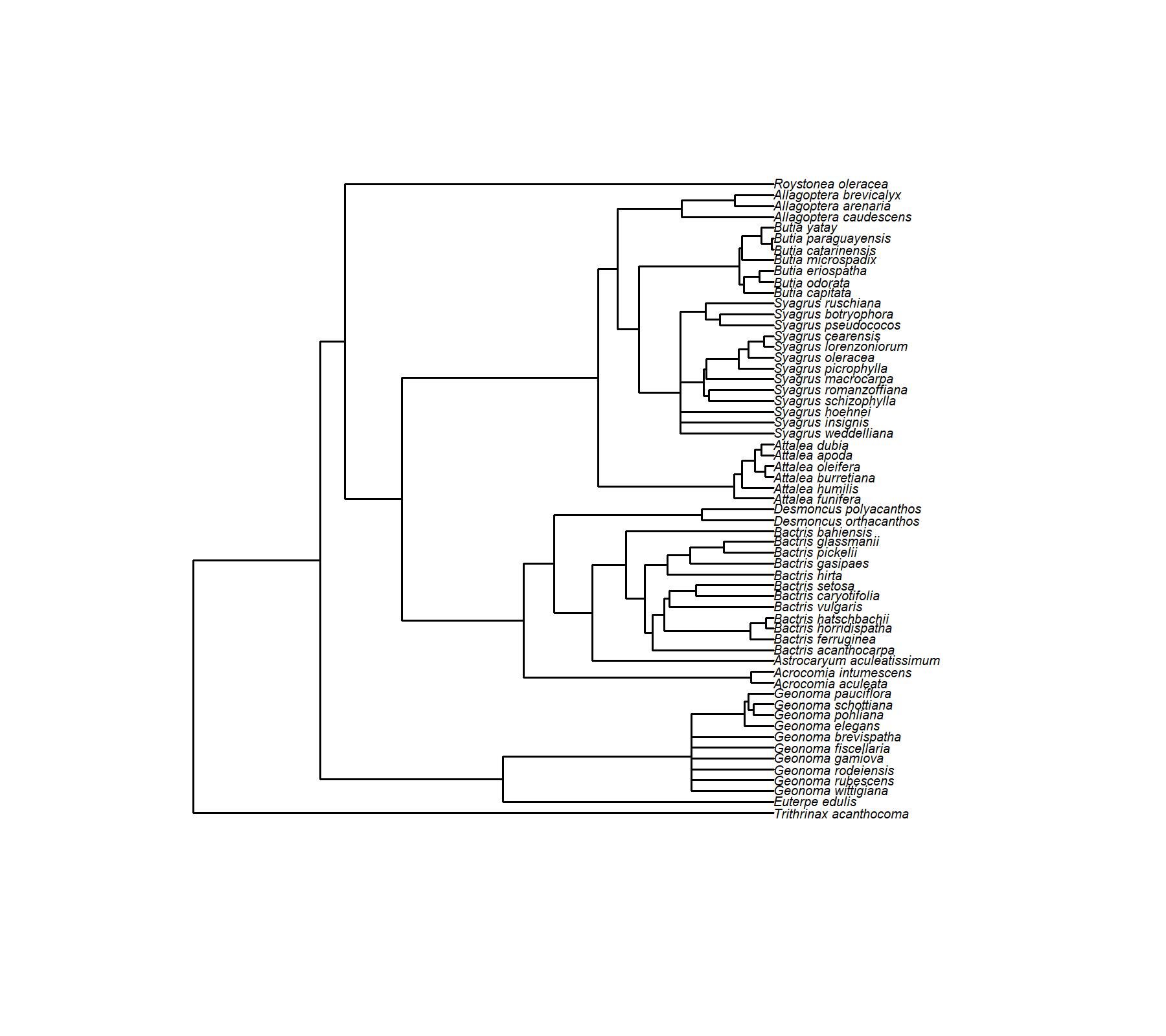
**

# Figure S1. Phylogenetic tree of the 59 palm species from the Atlantic Forest used in the PGLS analysis.

#

#

# Table S5. Model selection for the response variable: area change ratio under the optimistic 2050 scenario.

| **Rank** | **Model** | **df** | **AICc** | **delta** | **weight** |
| --- | --- | --- | --- | --- | --- |
| 1 | Ratio Optimistic 2050 ~ log Current Area | 2 | 55.86 | 0 | 0.354 |
| 2 | Ratio Optimistic 2050 ~ Habitat + log Current Area | 3 | 57.01 | 1.15 | 0.198 |
| 3 | Ratio Optimistic 2050 ~ log Current Area + Average Fruit Width | 3 | 57.91 | 2.05 | 0.127 |
| 4 | Ratio Optimistic 2050 ~ log Current Area + Max Stem Height | 3 | 58.00 | 2.14 | 0.121 |
| 5 | Ratio Optimistic 2050 ~ Habitat + log Current Area + Average Fruit Width | 4 | 59.15 | 3.29 | 0.064 |
| 6 | Ratio Optimistic 2050 ~ Habitat + log Current Area + Max Stem Height | 4 | 59.25 | 3.39 | 0.064 |
| 7 | Ratio Optimistic 2050 ~ log Current Area + Average Fruit Width + Max Stem Height | 4 | 60.18 | 4.31 | 0.040 |
| 8 | Ratio Optimistic 2050 ~ Habitat + log Current Area + Average Fruit Width + Max Stem Height | 5 | 61.52 | 5.65 | 0.020 |
| 9 | Ratio Optimistic 2050 ~ Habitat | 2 | 67.91 | 12.04 | 0.001 |
| 10 | Ratio Optimistic 2050 ~ Habitat + Max Stem Height | 3 | 68.27 | 12.40 | 0.001 |
| 11 | Ratio Optimistic 2050 ~ Habitat + Average Fruit Width | 3 | 69.19 | 13.32 | 0.000 |
| 12 | Ratio Optimistic 2050 ~ Max Stem Height | 2 | 69.20 | 13.34 | 0.000 |
| 13 | NULL | 1 | 69.43 | 13.56 | 0.000 |
| 14 | Ratio Optimistic 2050 ~ Habitat + Average Fruit Width + Max Stem Height | 4 | 70.16 | 14.30 | 0.000 |
| 15 | Ratio Optimistic 2050 ~ Average Fruit Width | 2 | 70.44 | 14.58 | 0.000 |
| 16 | Ratio Optimistic 2050 ~ Average Fruit Width + Max Stem Height | 3 | 70.95 | 15.09 | 0.000 |

#

# Table S6. Model selection for the response variable: area change ratio under the pessimistic 2050 scenario.

| **Rank** | **Model** | **df** | **AICc** | **delta** | **weight** |
| --- | --- | --- | --- | --- | --- |
| 1 | Ratio Pessimistic 2050 ~ log Current Area | 2 | 70.62 | 0 | 0.339 |
| 2 | Ratio Pessimistic 2050 ~ log Current Area + Habitat | 3 | 71.56 | 0.94 | 0.212 |
| 3 | Ratio Pessimistic 2050 ~ log Current Area + Max Stem Height | 3 | 72.63 | 2.02 | 0.123 |
| 4 | Ratio Pessimistic 2050 ~ log Current Area + Average Fruit Width | 3 | 72.82 | 2.20 | 0.112 |
| 5 | Ratio Pessimistic 2050 ~ log Current Area + Max Stem Height + Habitat | 4 | 73.63 | 3.01 | 0.075 |
| 6 | Ratio Pessimistic 2050 ~ Habitat + log Current Area + Average Fruit Width | 4 | 73.84 | 3.22 | 0.067 |
| 7 | Ratio Pessimistic 2050 ~ log Current Area + Average Fruit Width + Max Stem Height | 4 | 74.88 | 4.26 | 0.040 |
| 8 | Ratio Pessimistic 2050 ~ Habitat + log Current Area + Average Fruit Width + Max Stem Height | 5 | 75.96 | 5.34 | 0.023 |
| 9 | Ratio Pessimistic 2050 ~ Habitat | 2 | 81.02 | 10.41 | 0.001 |
| 10 | NULL | 1 | 82.75 | 12.13 | 0.000 |
| 11 | Ratio Pessimistic 2050 ~ Average Fruit Width + Habitat | 3 | 82.77 | 12.15 | 0.000 |
| 12 | Ratio Pessimistic 2050 ~ Habitat + Max Stem Height | 3 | 82.80 | 12.18 | 0.000 |
| 13 | Ratio Pessimistic 2050 ~ Max Stem Height | 2 | 84.14 | 13.52 | 0.000 |
| 14 | Ratio Pessimistic 2050 ~ Average Fruit Width | 2 | 84.26 | 13.64 | 0.000 |
| 15 | Ratio Pessimistic 2050 ~ Habitat + Average Fruit Width + Max Stem Height | 4 | 84.82 | 14.20 | 0.000 |
| 16 | Ratio Pessimistic 2050 ~ Average Fruit Width + Max Stem Height | 3 | 86.02 | 15.40 | 0.000 |

#

# Table S7. Model selection for the response variable: area change ratio under the optimistic 2070 scenario.

| **Rank** | **Model** | **df** | **AICc** | **delta** | **weight** |
| --- | --- | --- | --- | --- | --- |
| 1 | Ratio Optimistic 2070 ~ log Current Area | 2 | 69.69 | 0 | 0.382 |
| 2 | Ratio Optimistic 2070 ~ log Current Area + Habitat | 3 | 71.28 | 1.59 | 0.172 |
| 3 | Ratio Optimistic 2070 ~ log Current Area + Average Fruit Width | 3 | 71.74 | 2.05 | 0.136 |
| 4 | Ratio Optimistic 2070 ~ log Current Area + log Max Stem Height | 3 | 71.91 | 2.22 | 0.125 |
| 5 | Ratio Optimistic 2070 ~ Habitat + log Current Area + Average Fruit Width | 4 | 73.43 | 3.73 | 0.058 |
| 6 | Ratio Optimistic 2070 ~ Habitat + log Current Area + Max Stem Height | 4 | 73.58 | 3.89 | 0.054 |
| 7 | Ratio Optimistic 2070 ~ log Current Area + l Max Stem Height + Average Fruit Width | 4 | 74.03 | 4.34 | 0.043 |
| 8 | Ratio Optimistic 2070 ~ Habitat + log Current Area + Average Fruit Width + Max Stem Height | 5 | 75.80 | 6.10 | 0.018 |
| 9 | Ratio Optimistic 2070 ~ Habitat | 2 | 80.49 | 10.79 | 0.002 |
| 10 | NULL | 1 | 81.06 | 11.37 | 0.001 |
| 11 | Ratio Optimistic 2070 ~ Habitat + Max Stem Height | 3 | 81.65 | 11.96 | 0.001 |
| 12 | Ratio Optimistic 2070 ~ Max Stem Height | 2 | 81.79 | 12.10 | 0.000 |
| 13 | Ratio Optimistic 2070 ~ Average Fruit Width + Habitat | 3 | 81.84 | 12.14 | 0.000 |
| 14 | Ratio Optimistic 2070 ~ Average Fruit Width | 2 | 82.17 | 12.48 | 0.000 |
| 15 | Ratio Optimistic 2070 ~ Habitat + Average Fruit Width + Max Stem Height | 4 | 83.48 | 13.79 | 0.000 |
| 16 | Ratio Optimistic 2070 ~ Average Fruit Width + Max Stem Height | 3 | 83.48 | 13.79 | 0.000 |

#

# Table S8. Model selection for the response variable: area change ratio under the pessimistic 2070 scenario.

| **Rank** | **Model** | **df** | **AICc** | **delta** | **weight** |
| --- | --- | --- | --- | --- | --- |
| 1 | Ratio Pessimistic 2070 ~ log Current Area | 2 | 91.43 | 0 | 0.301 |
| 2 | Ratio Pessimistic 2070 ~ log Current Area + Max Stem Height | 3 | 92.53 | 1.10 | 0.172 |
| 3 | Ratio Pessimistic 2070 ~ Max Stem Height + Habitat | 3 | 92.74 | 1.31 | 0.155 |
| 4 | Ratio Pessimistic 2070 ~ log Current Area + Average Fruit Width | 3 | 93.60 | 2.17 | 0.101 |
| 5 | Ratio Pessimistic 2070 ~ log Current Area + Habitat + Max Stem Height | 4 | 93.87 | 2.43 | 0.088 |
| 6 | Ratio Pessimistic 2070 ~ log Current Area + Average Fruit Width + Max Stem Height | 4 | 94.84 | 3.41 | 0.054 |
| 7 | Ratio Pessimistic 2070 ~ log Current Area + Average Fruit Width + Habitat | 4 | 94.99 | 3.56 | 0.050 |
| 8 | Ratio Pessimistic 2070 ~ log Current Area + Habitat + Average Fruit Width + Max Stem Height | 5 | 96.26 | 4.83 | 0.026 |
| 9 | Ratio Pessimistic 2070 ~ Habitat | 2 | 97.42 | 5.99 | 0.015 |
| 10 | NULL | 1 | 97.99 | 6.56 | 0.011 |
| 11 | Ratio Pessimistic 2070 ~ log Current Area + Average Fruit Width | 3 | 99.59 | 8.16 | 0.005 |
| 12 | Ratio Pessimistic 2070 ~ Habitat + Max Stem Height | 3 | 99.63 | 8.20 | 0.004 |
| 13 | Ratio Pessimistic 2070 ~ Average Fruit Width | 2 | 100.02 | 8.59 | 0.004 |
| 14 | Ratio Pessimistic 2070 ~ Max Stem Height | 2 | 100.13 | 8.69 | 0.003 |
| 15 | Ratio Pessimistic 2070 ~ Habitat + Average Fruit Width + Max Stem Height | 4 | 101.86 | 10.43 | 0.001 |
| 16 | Ratio Pessimistic 2070 ~ Average Fruit Width + Max Stem Height | 3 | 102.24 | 10.81 | 0.001 |

# Table S9. Ratio of area change for each of the 59 palm species under future climate scenarios relative to the current (baseline) scenario. A value of 0 indicates complete loss of suitable area, 1 indicates no change, values greater than 1 indicate an expansion, and values between 0 and 1 indicate a contraction of suitable area under future climate conditions.

| **Species** | **Ratio optimistic 2050** | **Ratio pessimistic 2050** | **Ratio optimistic 2070** | **Ratio pessimistic 2070** |
| --- | --- | --- | --- | --- |
| *Acrocomia aculeata* | 1.02 | 1.01 | 1.04 | 1.04 |
| *Acrocomia intumescens* | 0.39 | 0.26 | 0.29 | 0.12 |
| *Allagoptera arenaria* | 1.00 | 1.00 | 1.01 | 1.00 |
| *Allagoptera brevicalyx* | 0.92 | 0.92 | 0.92 | 0.90 |
| *Allagoptera caudescens* | 0.98 | 0.99 | 0.97 | 1.00 |
| *Attalea apoda* | 0.19 | 0.09 | 0.08 | 0.06 |
| *Attalea burretiana* | 0.82 | 0.71 | 0.77 | 0.63 |
| *Attalea dubia* | 0.86 | 0.84 | 0.82 | 0.80 |
| *Attalea funifera* | 1.18 | 1.24 | 1.29 | 1.41 |
| *Attalea humilis* | 1.30 | 1.54 | 1.42 | 1.66 |
| *Attalea oleifera* | 0.50 | 0.40 | 0.40 | 0.16 |
| *Bactris acanthocarpa* | 1.00 | 0.99 | 1.00 | 0.99 |
| *Bactris bahiensis* | 1.20 | 1.40 | 1.32 | 1.66 |
| *Bactris caryotifolia* | 0.83 | 0.96 | 0.87 | 1.00 |
| *Bactris ferruginea* | 1.06 | 1.07 | 1.08 | 1.07 |
| *Bactris gasipaes* | 0.60 | 0.51 | 0.56 | 0.43 |
| *Bactris glassmanii* | 1.58 | 1.69 | 1.71 | 1.87 |
| *Bactris hatschbachii* | 0.93 | 0.95 | 0.94 | 0.98 |
| *Bactris hirta* | 0.99 | 0.97 | 0.98 | 0.97 |
| *Bactris horridispatha* | 0.79 | 0.69 | 0.81 | 0.64 |
| *Bactris pickelii* | 0.97 | 0.99 | 0.98 | 0.98 |
| *Bactris setosa* | 1.03 | 1.04 | 1.05 | 1.09 |
| *Bactris vulgaris* | 1.26 | 1.37 | 1.37 | 1.72 |
| *Butia capitata* | 0.99 | 0.97 | 0.97 | 0.98 |
| *Butia catarinensis* | 1.04 | 1.07 | 1.08 | 1.12 |
| *Butia eriospatha* | 0.91 | 0.90 | 0.90 | 0.84 |
| *Butia microspadix* | 0.62 | 0.60 | 0.43 | 0.81 |
| *Butia odorata* | 1.00 | 1.00 | 1.00 | 1.00 |
| *Butia paraguayensis* | 1.25 | 1.28 | 1.29 | 1.30 |
| *Butia yatay* | 0.99 | 0.99 | 0.98 | 0.96 |
| *Desmoncus orthacanthos* | 1.06 | 1.07 | 1.07 | 1.11 |
| *Desmoncus polyacanthos* | 1.01 | 1.05 | 1.04 | 1.10 |
| *Euterpe edulis* | 0.94 | 0.90 | 0.93 | 0.85 |
| *Geonoma brevispatha* | 0.55 | 0.48 | 0.49 | 0.32 |
| *Geonoma elegans* | 0.91 | 0.91 | 0.90 | 0.89 |
| *Geonoma fiscellaria* | 0.86 | 0.80 | 0.92 | 1.13 |
| *Geonoma gamiova* | 0.88 | 0.71 | 0.79 | 0.53 |
| *Geonoma pauciflora* | 0.90 | 0.89 | 0.89 | 0.85 |
| *Geonoma pohliana* | 0.73 | 0.68 | 0.70 | 0.60 |
| *Geonoma rodeiensis* | 0.95 | 0.99 | 0.99 | 0.99 |
| *Geonoma rubescens* | 1.89 | 1.95 | 1.95 | 2.01 |
| *Geonoma schottiana* | 0.80 | 0.75 | 0.78 | 0.72 |
| *Geonoma wittigiana* | 0.55 | 0.60 | 0.50 | 0.38 |
| *Roystonea oleracea* | 0.95 | 0.92 | 0.94 | 0.86 |
| *Syagrus botryophora* | 0.90 | 0.84 | 0.87 | 0.76 |
| *Syagrus cearensis* | 0.52 | 0.44 | 0.46 | 0.34 |
| *Syagrus hoehnei* | 0.85 | 0.76 | 0.83 | 0.72 |
| *Syagrus insignis* | 0.83 | 0.75 | 0.70 | 0.89 |
| *Syagrus lorenzoniorum* | 0.04 | 0.01 | 0.08 | 0.00 |
| *Syagrus macrocarpa* | 1.05 | 1.07 | 1.06 | 1.07 |
| *Syagrus oleracea* | 0.97 | 0.95 | 0.97 | 0.95 |
| *Syagrus picrophylla* | 0.94 | 0.88 | 0.91 | 0.85 |
| *Syagrus pseudococos* | 0.84 | 0.79 | 0.82 | 0.73 |
| *Syagrus romanzoffiana* | 0.99 | 0.99 | 0.99 | 0.97 |
| *Syagrus ruschiana* | 1.46 | 1.46 | 1.46 | 1.46 |
| *Syagrus schizophylla* | 0.91 | 0.88 | 0.90 | 0.85 |
| *Syagrus weddelliana* | 0.92 | 0.87 | 0.89 | 0.87 |
| *Trithrinax acanthocoma* | 0.94 | 0.92 | 0.91 | 0.83 |

**Figure S2.** The graphs illustrate the percentage of area lost (red) and the percentage of area gained (blue) in each future climate change scenario, for each palm species.

(a) Optimistic 2050


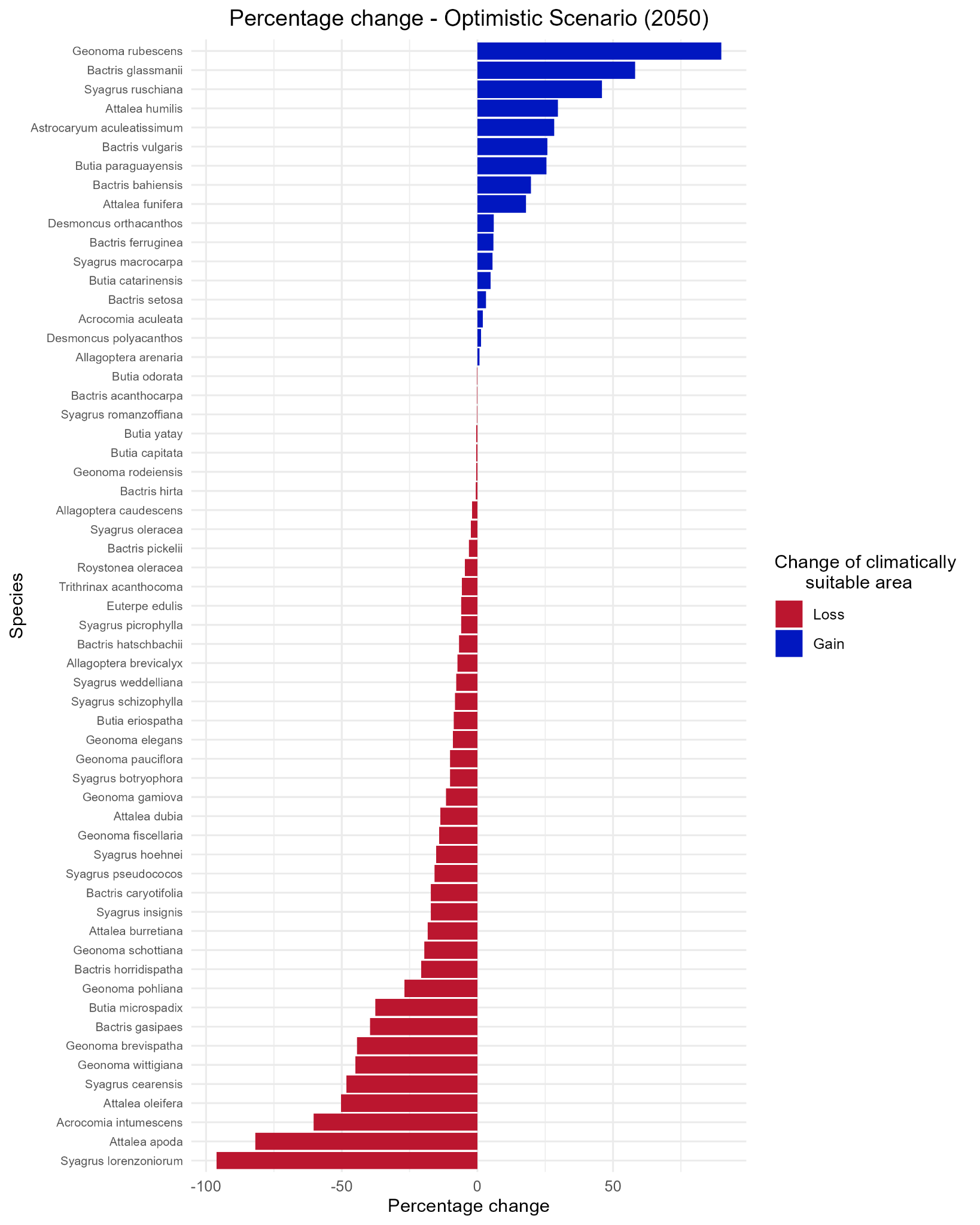


(b) Pessimistic 2050


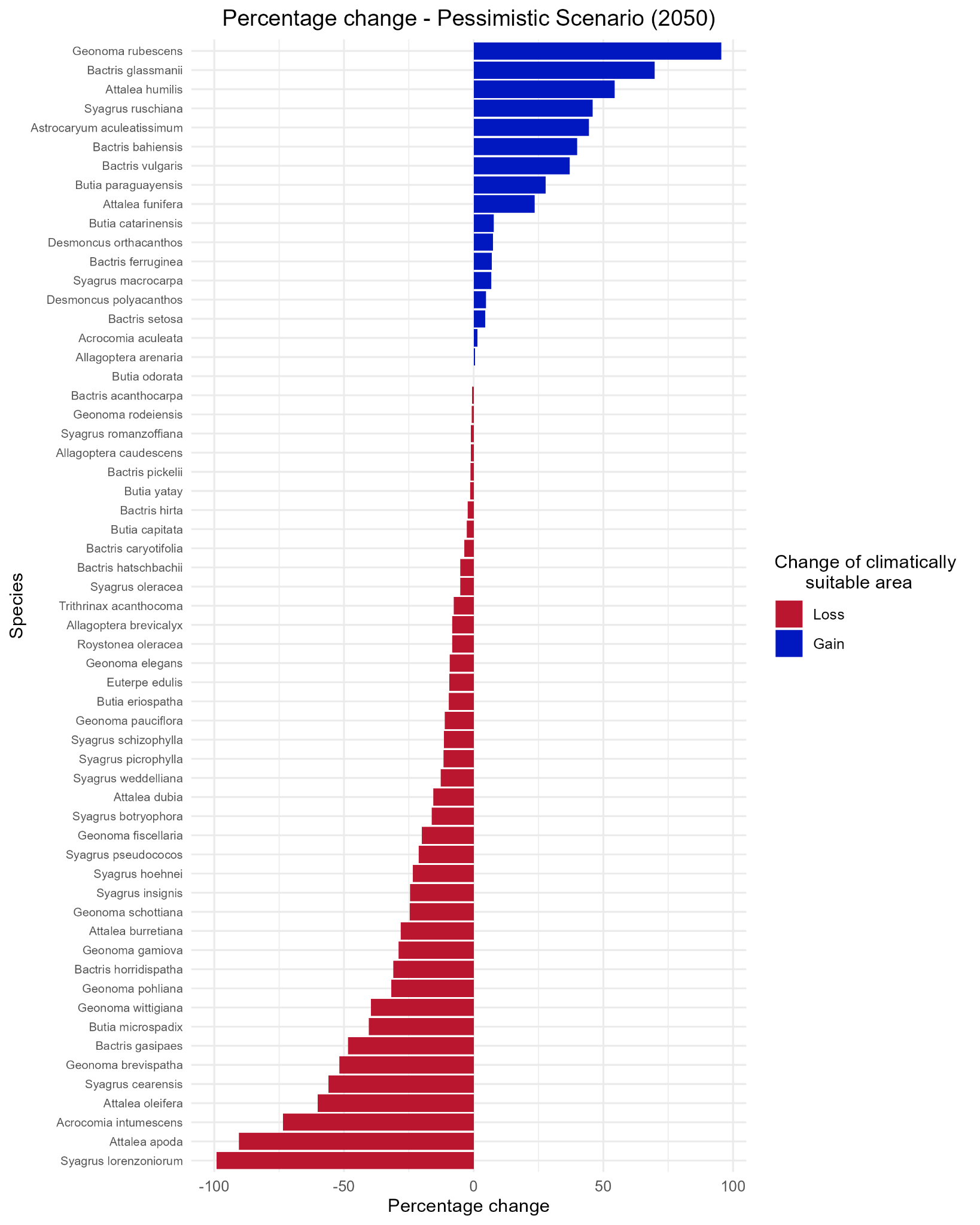


(c) Optimistic 2070


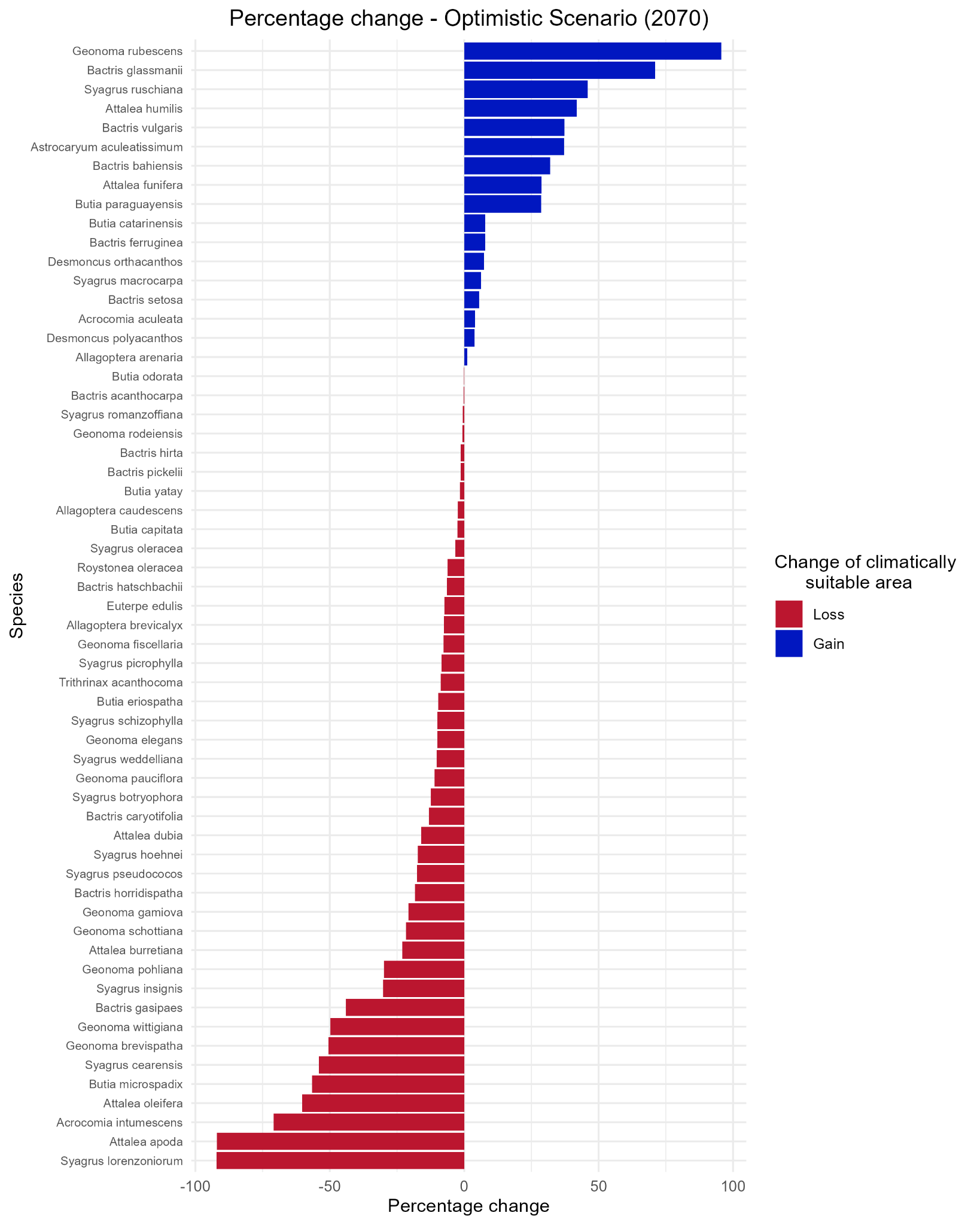


(d) Pessimistic 2070


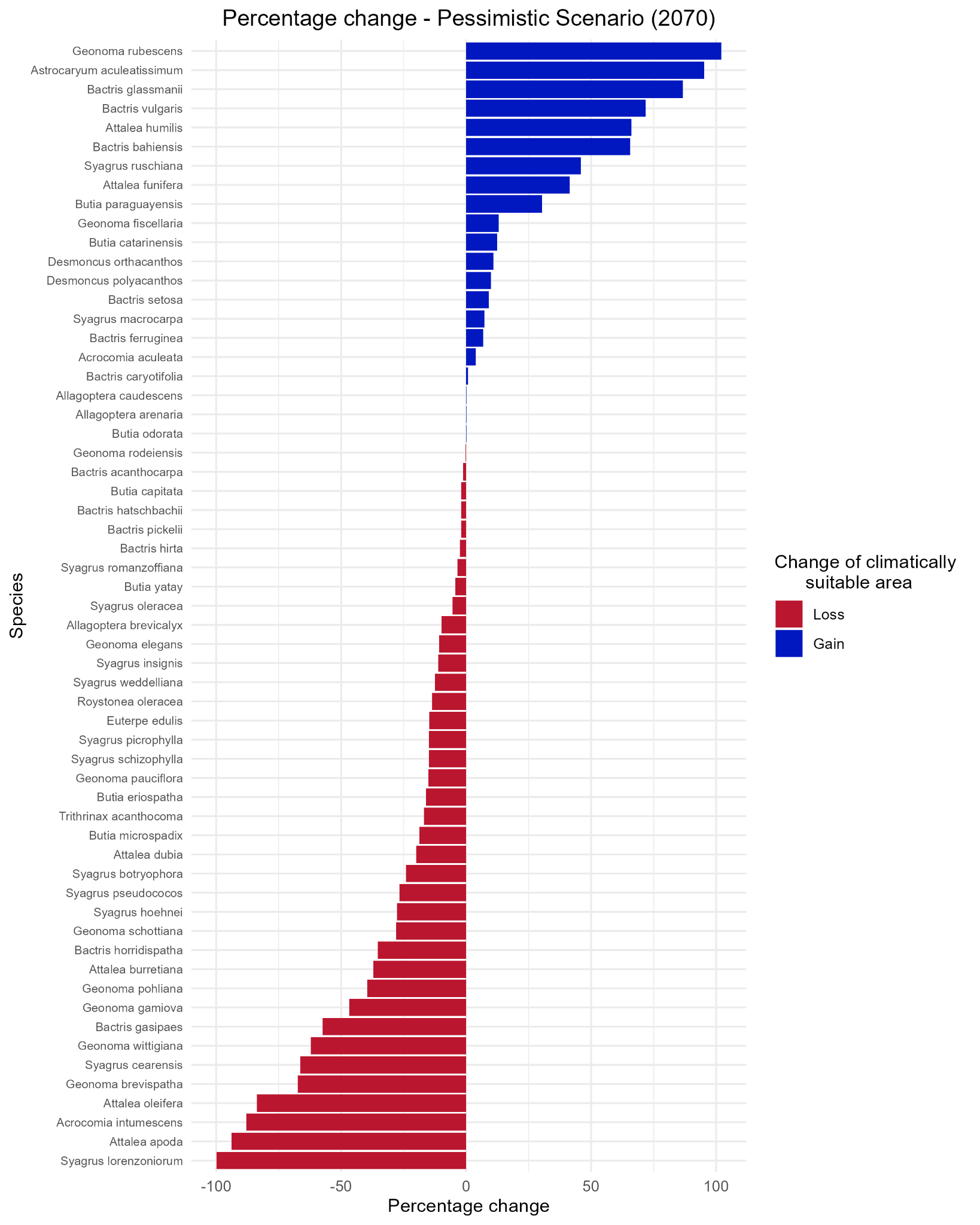


#

# Supplementary references

Bello, C., Galetti, M., Montan, D., Pizo, M.A., Mariguela, T.C., Culot, L., Bufalo, F., Labecca, F., Pedrosa, F., Constantini, R., Emer, C., Silva, W.R., Da Silva, F.R., Ovaskainen, O., Jordano, P., 2017. “Atlantic frugivory: a plant–frugivore interaction data set for the Atlantic Forest.” *Ecology* 98, 1729–1729. https://doi.org/10.1002/ecy.1818

Blach‐Overgaard, A., Svenning, J., Dransfield, J., Greve, M., Balslev, H., 2010. “Determinants of palm species distributions across Africa: the relative roles of climate, non‐climatic environmental factors, and spatial constraints.” *Ecography* 33, 380–391. https://doi.org/10.1111/j.1600-0587.2010.06273.x

Blach-Overgaard, A., Svenning, J.-C., Balslev, H., 2009. “Climate change sensitivity of the African ivory nut palm, *Hyphaene petersiana* Klotzsch ex Mart. (Arecaceae) – a keystone species in SE Africa.” *IOP Conf. Ser.: Earth Environ. Sci*. 8, 012014. https://doi.org/10.1088/1755-1315/8/1/012014

Cerqueira, A.F., Benchimol, M., Sousa‐Santos, C., Bezerra, I.M., Santana dos Santos, M., Dalmolin, Â.C., Gaiotto, F.A., Mielke, M.S., 2023. “Trends and gaps in the literature on native palms of the Brazilian Atlantic Forest.” *Austral Ecology* 49, e13322. https://doi.org/10.1111/aec.13322

Heming, N. M.; Dambros, C.; Gutiérrez, E. E., 2019. “ENMwizard: AIC model averaging and other advanced techniques in Ecological Niche Modeling made easy.” (Version 0.4.2) [R Package]. https://github.com/HemingNM/ENMwizard.

Hernandez, P.A., Graham, C.H., Master, L.L., Albert, D.L., 2006. “The effect of sample size and species characteristics on performance of different species distribution modeling methods.” *Ecography* 29, 773–785. https://doi.org/10.1111/j.0906-7590.2006.04700.x

Lorenzi, H., Noblick, L., Kahn, F., Ferreira, E.J.L., 2010. “Flora Brasileira: Arecaceae (Palmeiras).” Instituto Plantarum, Nova Odessa, SP.

Muscarella, R., Galante, P.J., Soley‐Guardia, M., Boria, R.A., Kass, J.M., Uriarte, M., Anderson, R.P., 2014. “ENM eval: An R package for conducting spatially independent evaluations and estimating optimal model complexity for Maxent ecological niche models.” *Methods in Ecology and Evolution* 5, 1198–1205. https://doi.org/10.1111/2041-210X.12261

Phillips, S.J., Anderson, R.P., Schapire, R.E., 2006. "Maximum entropy modeling of species geographic distributions.” *Ecological Modelling* 190, 231–259. https://doi.org/10.1016/j.ecolmodel.2005.03.026

Phillips, S.J., Anderson, R.P., Dudík, M., Schapire, R.E., Blair, M.E., 2017. “Opening the black box: an open‐source release of Maxent.” *Ecography* 40, 887–893. https://doi.org/10.1111/ecog.03049
